# Reconstruction and optimization of a *Pseudomonas putida*-*Escherichia coli* microbial consortium for mcl-PHA production from lignocellulosic biomass

**DOI:** 10.3389/fbioe.2022.1023325

**Published:** 2022-10-19

**Authors:** Ruolin Qin, Yinzhuang Zhu, Mingmei Ai, Xiaoqiang Jia

**Affiliations:** ^1^ Department of Biochemical Engineering, School of Chemical Engineering and Technology, Tianjin University, Tianjin, China; ^2^ Collaborative Innovation Center of Chemical Science and Engineering (Tianjin), Tianjin, China; ^3^ Frontier Science Center for Synthetic Biology and Key Laboratory of Systems Bioengineering (MOE), School of Chemical Engineering and Technology, Tianjin University, Tianjin, China

**Keywords:** artificial microbial consortium, MCL-PHA, engineered *Escherichia coli*, engineered *Pseudomonas putida*, lignocellulosic hydrolysate

## Abstract

The demand for non-petroleum-based, especially biodegradable plastics has been on the rise in the last decades. Medium-chain-length polyhydroxyalkanoate (mcl-PHA) is a biopolymer composed of 6–14 carbon atoms produced from renewable feedstocks and has become the focus of research. In recent years, researchers aimed to overcome the disadvantages of single strains, and artificial microbial consortia have been developed into efficient platforms. In this work, we reconstructed the previously developed microbial consortium composed of engineered *Pseudomonas putida* KT∆ABZF (p2-a-J) and *Escherichia coli* ∆4D (ACP-SCLAC). The maximum titer of mcl-PHA reached 3.98 g/L using 10 g/L glucose, 5 g/L octanoic acid as substrates by the engineered *P. putida* KT∆ABZF (p2-a-J). On the other hand, the maximum synthesis capacity of the engineered *E. coli* ∆4D (ACP-SCLAC) was enhanced to 3.38 g/L acetic acid and 0.67 g/L free fatty acids (FFAs) using 10 g/L xylose as substrate. Based on the concept of “nutrient supply-detoxification,” the engineered *E. coli* ∆4D (ACP-SCLAC) provided nutrient for the engineered *P. putida* KT∆ABZF (p2-a-J) and it acted to detoxify the substrates. Through this functional division and rational design of the metabolic pathways, the engineered *P. putida*-*E. coli* microbial consortium could produce 1.30 g/L of mcl-PHA from 10 g/L glucose and xylose. Finally, the consortium produced 1.02 g/L of mcl-PHA using lignocellulosic hydrolysate containing 10.50 g/L glucose and 10.21 g/L xylose as the substrate. The consortium developed in this study has good potential for mcl-PHA production and provides a valuable reference for the production of high-value biological products using inexpensive carbon sources.

## 1 Introduction

The diminishing supply of fossil resources and ecological problems caused by petroleum-based plastics have led to a new wave of exploration of biodegradable polymers that can replace traditional petroleum-based plastics (Koller and Mukherjee, 2022). Polyhydroxyalkanoates (PHAs) are natural polyesters that can be synthesized by microorganisms from renewable sources (Chen et al., 2020). PHAs are considered a viable alternative to petroleum-based plastics due to their excellent biodegradability, biocompatibility, optical activity, gas separation, piezoelectricity, and general physiochemical properties similar to petroleum-based plastics (Wang et al., 2015; Tarrahi et al., 2020).

The material properties of PHAs depend on the type and distribution of the various monomeric structural units, which can be classified into three groups on the basis of the number of constituent carbon atoms, including short-chain-length polyhydroxyalkanoates (scl-PHAs) such as PHB, composed of 3-5 carbon atoms, medium-chain-length polyhydroxyalkanoates (mcl-PHAs), consisting of monomers with 6–14 carbon atoms, and long-chain-length polyhydroxyalkanoates (lcl-PHAs), consisting of monomers with more than 15 carbon atoms (Xiang et al., 2020; Behera et al., 2022). There are different degrees of variation in the crystallinity and tensile strength of PHAs as the chain length changes (Elmowafy et al., 2019). Mcl-PHAs have a lower glass transition temperature (Tg) and melting point (Tm) than scl-PHAs (Rai et al., 2011; Gopi et al., 2018). This makes mcl-PHAs thermo-elastic, and once the temperature rises above the Tm value, mcl-PHAs exhibit characteristics such as softness, flexibility and amorphous behavior (Grigore et al., 2019). Accordingly, mcl-PHA are considered true elastomers, whereas most scl-PHAs are hard and friable highly crystalline polymers (Knoll et al., 2009). Due to this structural diversity, mcl-PHAs can meet the flexible demands of a wider range of engineering applications (Chen and Wang, 2013; Zhang et al., 2015). However, the industrial production of mcl-PHAs is currently negligible compared to the production and commercialization of scl-PHAs (Koller and Mukherjee, 2022).

The current barriers limiting the large-scale industrial production of mcl-PHAs are mainly the process cost and production capacity (Mannina et al., 2020; De Donno Novelli et al., 2021). The currently available microbial cell factories require the provision of expensive precursors such as FFAs and have limited production capacity. We therefore need to better understand which natural microorganisms are suitable for mcl-PHA production from a given substrate and how to modify natural microorganisms by means of metabolic engineering to develop efficient cell factories, which will enable us to reduce production costs by using inexpensive carbon sources.

Mcl-PHAs can be produced naturally by a variety of microorganisms, especially species of *Pseudomonas* (Możejko-Ciesielska and Kiewisz, 2016; Chio et al., 2019). For example, *P. putida* KT2440 can naturally produce mcl-PHA as an energy storage material under conditions of surplus FFAs. Moreover, it has a clear genetic background and diverse metabolism pathways. The synthesis of mcl-PHAs in *P. putida* KT2440 proceeds through the fatty acid β-oxidation pathway and is based on so-called related carbon sources such as FFAs. However, it can also use the fatty acid *de novo* pathway to produce mcl-PHAs from unrelated carbon sources such as sugars (Prieto et al., 2016). There are two key enzymes that link the fatty acid β-oxidation pathway to mcl-PHA biosynthesis. The enzyme encoded by the *phaJ* gene converts the β-oxidation intermediate enoyl-CoA into 3-hydroxyacyl-CoA (Belda et al., 2016), which is then polymerized into mcl-PHA by the enzyme encoded by *phaC*. Furthermore, in terms of metabolic pathways, direct provision of FFAs seems to be an effective strategy for enhancing mcl-PHA, but as mentioned above this also results in a significant increase of process cost, thus requiring us to look for less expensive carbon sources. A good candidate is lignocellulose, which is widely available and considered to be the most abundant organic raw material on earth (Al-Battashi et al., 2019). In the past decades, the conversion of lignocellulosic biomass into value-added products has attracted increasing attention, and has been widely studied due to its low cost and other advantages. However, lignocellulose contains a mixture of sugars that make it difficult for a single strain to fully utilize the available carbon source, and although metabolic engineering may give them the ability to utilize multiple substrates (Wang et al., 2018), it also increases the metabolic burden. On top of that, pure cultures have high maintenance costs, which runs counter to the starting point of using lignocellulose to reduce costs (Chen et al., 2019). Considering these problems, researchers tried to produce mcl-PHA using natural microbial communities, but the functions of the individual strains in these communities could not be fully determined. Moreover, the time required to domesticate wild strains is long, and their transformation efficiency is generally low (Morgan-Sagastume et al., 2015). In order to solve this problem, the targeted design and construction of artificial microbial consortia from the perspective of synthetic biology development can achieve tasks that cannot be accomplished by pure cultures of individual microorganisms. Compared with natural communities, artificial microbial consortia with simple composition and clear division of labor can reduce the metabolic burden of monocultures and theoretically achieve efficient production of mcl-PHA by regulating the interactions between bacteria (Minty et al., 2013). However, the complexity of the design and construction of artificial microbial consortia has limited the achievable titers of mcl-PHA (Kataria et al., 2018).

In 2019, we strengthened the acetate assimilation pathway of *P. putida* KT2440 by overexpressing the *acs* gene encoding acetyl CoA synthase and constructing the acetate kinase-phosphotransacetylase pathway encoded by *ackA-pta* (Yang et al., 2019). The engineered *P. putida* produced 0.674 g/L of mcl-PHA from acetate, suggesting that acetate may be a potential substrate for the production of mcl-PHAs. To avoid the limitations of single-strain cultures, we constructed a microbial consortium consisting of engineered *E. coli* and *P. putida* based on the concept of “nutrient supply-detoxification” in 2020. Based on the engineered *P. putida* overexpressing the *acs* gene, the *ptsG* and *manZ* genes were knocked out in *E. coli* to reduce substrate competition so that it could preferentially utilize xylose, while the *atpFH* and *envR* genes were further knocked out to promote the ability of engineered *E. coli* to synthesize acetate and FFAs as precursors (Liu et al., 2020). When grown on a mixture of glucose and xylose, the consortium produced an increased mcl-PHA titer of 0.541 g/L, demonstrating its potential to utilize lignocellulose. However, the titer of mcl-PHA still needed to be improved due to the limited modification of engineered *P. putida*. To address this issue, we further optimized *P. putida* to improve the conversion efficiency of FFAs to mcl-PHA by knocking out the *fadA* and *fadB* genes in the FFAs β-oxidation pathway and overexpressing the *phaJ* gene (Zhu et al., 2021). At the same time, fatty acid catabolism was downregulated by knocking out the *fadD* gene and expressing a heterologous gene encoding the acyl carrier protein thioesterase from *E. coli*. The substrate conversion efficiency and mcl-PHA titer of the reconstituted microbial consortium were significantly improved, with a maximum mcl-PHA titer of 1.32 g/L obtained from a mixture of glucose and xylose. However, the functional validation of lignocellulose utilization by the consortium was limited to the use of a mixed carbon source comprising glucose and xylose, while the substrate conversion efficiency and productivity still needed further improvement.

**FIGURE 1:**
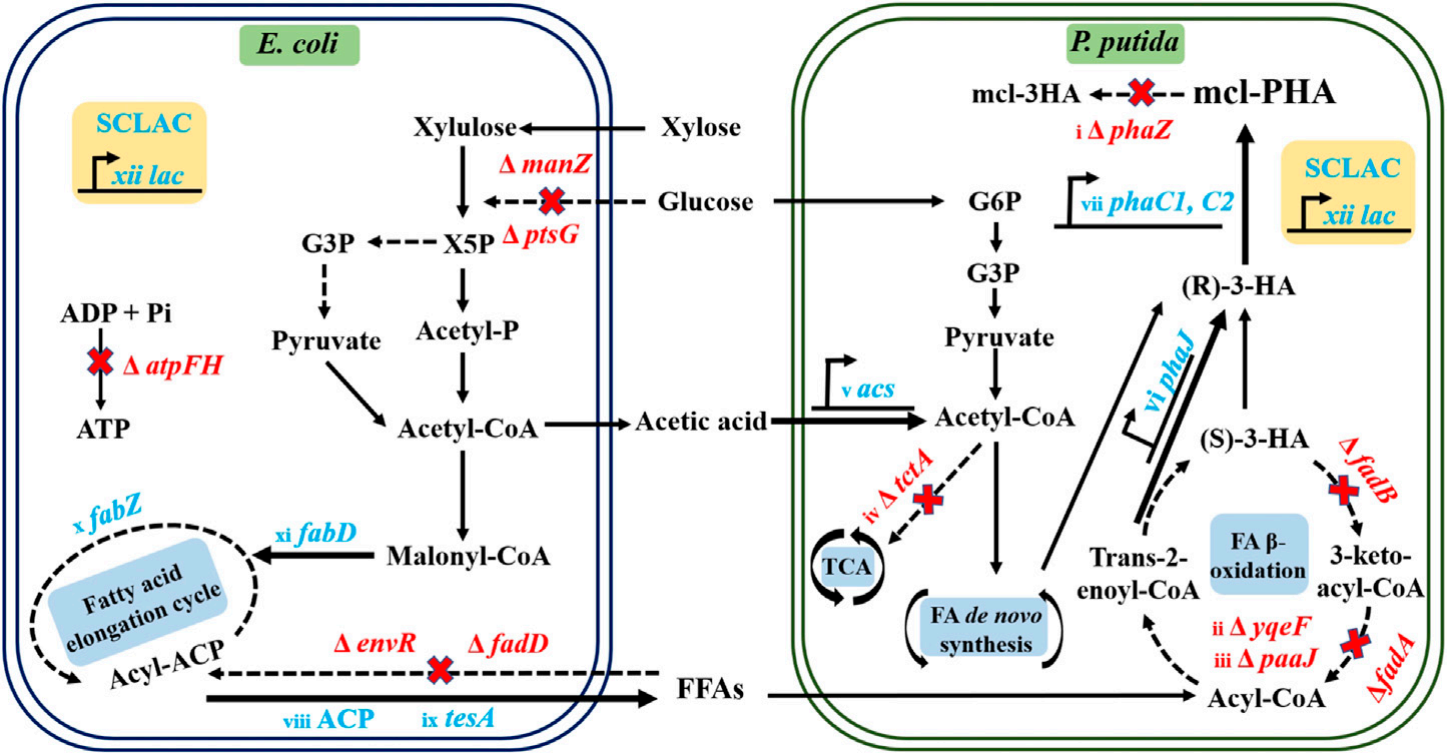
Schematic diagram of the metabolic engineering protocol for the production of mcl-PHA by the *P. putida*-*E. coli* microbial consortium. Acronyms: X5P, Xylulose 5-phosphate; G3P, Glyceraldehyde-3-phosphate; Acetyl-P, Acetyl Phosphate; G6P, Glucose-6-Phosphate. The red words in the biosynthetic pathway indicate the deletion of corresponding genes. The blue words indicate the overexpression of corresponding genes. The related genetic engineering represented by i∼xii was conducted in this work.

In this study, we further enhanced the metabolic functions of the two strains in the artificial *P. putida*-*E. coli* microbial consortium (Figure 1), allowing further improvement in the conversion of substrates to intermediate metabolites and final products. This was achieved by introducing dual vectors encoding the acyl carrier protein thioesterase gene (*ACP*) and the *SCLAC* gene encoding laccase from *Streptomyces azureus* to improve the ability of the engineered *E. coli* to secrete acetic acid and FFAs, which were the material basis for interspecies communication. In the engineered *P. putida*, we further knocked out the *phaZ* gene encoding PHA depolymerase to restrict PHA catabolism and further knocked out the *yqeF* gene to weaken the fatty acid β-oxidation pathway, thus enhancing PHA precursor synthesis. In addition, we overexpressed the *phaJ* gene encoding enoyl coenzyme A hydratase and the *acs* gene in the acetic acid assimilation pathway to improve the efficiency of acetic acid and FFAs conversion. Based on this design, we reconstructed the artificial consortium, then optimized its aerobic fermentation conditions, including the proportion of mixed sugars, the balance of nutrient restriction, and cell growth. Finally, we tested the ability of the consortium to produce mcl-PHA using lignocellulosic hydrolysate.

## 2 Materials and methods

### 2.1 Bacterial strains, plasmids, and reagents

The strains and plasmids used in this study are summarized in Supplementary Table S1. The original strain *E. coli* MG1655 (ATCC 700926) was donated by Dr. Tao Chen of Tianjin University, and *E. coli* ∆4D was stored in our laboratory. The original strain *P. putida* KT2440 (ATCC 47054) was obtained from the American type culture collection (Manassas, VA, United States). *E. coli* S17-1 was stored in our laboratory. The plasmid pBBR1MCS-2 was donated by Dr. Yingjin Yuan of Tianjin University, China. The plasmids pTKS/CS and pTKRED were donated by Dr. Tao Chen of Tianjin University. The plasmids pET28a and pK18mobsacB were stored in our laboratory. Xylose (99% purity) was purchased from Yuanye (Shanghai, China). Acetate (HPLC grade) was purchased from Concord Tech (China). Glucose (AR) and P-coumaric acid (>98% purity) were purchased from Yuanli Chemical (Tianjin, China).

DNA manipulating agents, including restriction endonucleases and T4 DNA ligase, were purchased from Thermo Scientific (Beijing, China). Phanta Max Super-Fidelity DNA polymerase and Taq for polymerase chain reaction (PCR) were purchased from Vazyme (Nanjing, China). PCR primers were synthesized by Tsingke Biotechnology Co., Ltd. (Tianjin, China) and are summarized in Supplementary Table S2.

### 2.2 Plasmids and strains construction

*E. coli* MG1655, *E. coli* ∆4D and *E. coli* Δ4D (ACP) derivatives were used to construct the engineered strain for the biosynthesis of acetic acid and FFAs. The plasmid pET28a was used as a vector to construct a heterologous expression vector using the T3 promoter or Tac promoter to induce the expression of genes involved in the fatty acid synthesis pathway. The primers pET-T_3_-RBS-f/T_3_-RBS-f2/T_3_-RBS-f1/RBS-*tesA*-f and pET-*tesA*-r were used to amplify T_3_-RBS-*tesA* fragment using the *E. coli* MG1655 genome as a template. Similarly, the T_3_-RBS-*fabZ* fragment and the T3-RBS-*fabD* fragment were amplified by the same method using the corresponding primers, respectively. The front end and the end of the amplified fragment each contain a homologous arm linked to pET28a. The DH5α strain containing the pET28a plasmid was expanded and then the pET28a plasmid was extracted. The empty plasmid was double digested and the linear vector was recovered by agarose gel after double digestion, and the pET-*tesA*, pET-*fabZ* and pET-*fabD* vectors were constructed by seamless cloning with the above fragments. Then the overexpressed strains *E. coli* 4D (*tesA*), *E. coli* 4D (*fabZ*) and *E. coli* 4D (*fabD*) were obtained by electrotransformation, respectively. The overexpression vectors pET-*ACP*-*fabD*, pET- *ACP* -*tesA*-*fabD*, pET- *ACP* -*fabZ*-*fabD* and pET- *ACP* -*tesA*-fabZ-*fabD* were constructed by combining the above fatty acid-related genes with *ACP* based on the laboratory’s constructed expression vector pET- *ACP* that heterologously expresses the thioesterase gene of the ricin acyl carrier protein. Using the XbaI-*tesA*-f/NheI-*tesA*-r, NheI-*fabZ*-f/BamHI-*fabZ*-r and BamHI-*fabD*-f/NotI-*fabZ*-r primers, the three overexpression vectors constructed were used as templates to amplify the T3-RBS*-tesA* fragment, T3-RBS-*fabZ* fragment and T3-RBS-*fabD* fragment containing the corresponding enzyme cut sites, respectively. The pET- *ACP* plasmid and the T3-RBS-*fabD* fragment were double digested, and the linear vector and the enzyme fragment containing the sticky end were recovered by agarose gel, and the pET- *ACP* -*fabD* vector was constructed by ligation with T_4_ ligase. The plasmid was then transferred into *E. coli* Δ4D by electrotransformation and the positive overexpression strain *E. coli* Δ4D (AD) containing the overexpression vector was screened on LB plates containing Kanamycin. The pET- *ACP* -*tesA*-*fabD* vector, pET- *ACP* -*fabZ*-*fabD* vector and pET- *ACP* -*tesA-fabZ-fabD* vector were constructed using the same method and overexpression strains *E. coli* Δ4D (AAD), *E. coli* Δ4D (AZD) and *E. coli* Δ4D (AAZD) were obtained by electrotransformation.

*P. putid*a KT2440 was used to construct the engineered strain for mcl-PHA synthesis, acetic acid, and FFAs utilization. Based on our previous studies of co-expression of *acs* and *phaJ* genes, the RBS-*acs*-T_3_-RBS-*phaJ* fragment was amplified using the *acs*-*phaJ*-f/r primers and then the RBS-*acs*-T_3_-RBS-*phaJ* fragment was recovered by agarose gel. In addition, the DH5α strain containing the pBBR1MCS-2 plasmid was expanded and the pBBR1MCS-2 plasmid was extracted. The pBBR1MCS-2 plasmid and the RBS-*acs*-T_3_-RBS-*phaJ* fragment were subjected to double digestion in the PCR reaction system, the linear vector and the RBS-*acs*-T3-RBS-*phaJ* fragment with sticky ends were recovered by agarose gel after double digestion. Finally, the p2-*acs*-*phaJ* was obtained by ligating the vector and fragment using T4 ligase. Using the *P. putida* KT2440 genome as a template, the *phaC1* gene and the *phaC2* gene were amplified using the *phaC1*-f/r and *phaC2*-f/r primers, respectively, for the construction of the p2_Tac_-*C1C2* vector, the front and end of the amplified phaC1 fragment contain a homologous arm linked to the Tac-RBS fragment and a homologous arm linked to the phaC2 fragment, respectively. For the construction of the p2_T3_-*C1C2* vector, the front of the amplified RBS-*phaC1* fragment and the end of the *phaC1* fragment contain a homologous arm linked to the pBBR1MCS-2 and a homologous arm linked to the *phaC2* fragment, respectively. The front and end of the amplified *phaC2* fragment contain a homology arm linked to the *phaC1* fragment and a homology arm linked to the pBBR1MCS-2, respectively. The amplified fragments were recovered and then seamlessly clonally ligated to construct the Tac-RBS-*phaC1-phaC2* fragment and the RBS-*phaC1-phaC2* fragment. The pBBR1MCS-2 plasmid and RBS-*acs*-T_3_-RBS-phaJ fragment were double digested by PCR reaction system. The linear vector after double digestion was recovered by agarose gel, and the p2_Tac_-*C1C2* vector and p2_T3_-*C1C2* vector were constructed by seamless cloning ligation and then, as described before positive overexpression strains containing the p2_Tac_-*C1C2* vector and the p2_T3_-*C1C2* vector were obtained by electrotransformation.

### 2.3 Culture medium and growth conditions

Luria Bertani (LB) medium was used for strain preservation and seed culture preparation. The composition of the M9 medium in this work contained 12.8 g/L Na_2_HPO_4_ 7H_2_O, 3 g/L KH_2_PO_4_, 1 g/L NH_4_Cl, 0.5 g/L NaCl, and 0.24 g/L MgSO_4_, which was autoclaved and subsequently supplement with the required content of filter-sterilized glucose and/or xylose, etc., and a trace element solution containing 2.78 g/L FeSO_4_·7H_2_O, 1.98 g/L MnCl_2_·4H_2_O, 2.38 g/L CoCl_2_·6H_2_O, 1.47 g/L CaCl_2_·2H_2_O, 0.17 g/L CuCl_2_·2H_2_O, 0.29 g/L ZnSO_4_·7H_2_O. Where appropriate, 50 μg/ml kanamycin, 100 μg/ml chloramphenicol, 50 μg/ml streptomycin and 2 mM Isopropyl β-d-1-thiogalactopyranoside (IPTG) were added. For the shake flask fermentations, single colonies of *E. coli* and *P. putida* were grown in 5 ml of culture medium in test tubes overnight at 30°C and 220 rpm. The overnight cultures were used to inoculate 500 ml shake flasks containing 50 ml of M9 medium at a seed ratio of 1%, and fermented at 30°C and 220 rpm. For bioreactor fermentation, if not specified, the 500 ml conical flask with 50 ml of M9 medium was spiked with the appropriate concentration of carbon source, and the activated bacterial culture was inoculated in the medium at a 2% inoculation ratio and fermentation for 64 h at 30°C and 220 rpm. The fermentation experiments were conducted in triplicates, and data were shown as the mean values ± standard deviations (SD).

### 2.4 Acid-pretreatment and enzymatic digestion method of corn straw

The corn straw pretreatment solution was donated by Dr. Bingzhi Li of Tianjin University, China. The specific pretreatment method was: the collected corn straw was crushed and sieved and washed twice using distilled water, the straw pellets between 20 and 80 mesh were collected, dried and set aside. The corn straw pellets (10%, w/v) were placed in a blue-capped bottle containing distilled water and pretreated with a dose of sulphuric acid 1% (v/v) and mixed thoroughly. The blue-capped bottle was heated to 130°C in an autoclave and held for 30 min. Then the bottle was then dried to room temperature and the solution was collected by shaking dry and dehydrated as corn stover pretreatment solution. The solid residue was removed by freezing and centrifuging the corn stover pretreatment solution at 9,000 rpm for 10 min. The pH was adjusted to approximately 7.0 with NaOH and the sugar content of the pretreatment solution was analyzed by HPLC.

The enzymatic reaction was carried out using cellulase and hemicellulase enzymes to obtain the corn stover digestion using the solid corn stover pellet powder pretreated with acid as a reaction substrate as described above. The reaction was carried out as follows: the corn stover pellet powder was dried at room temperature, placed in a conical flask containing sodium citrate buffer (0.05 M, pH4.8) and enzymatically digested with cellulase and hemicellulase (mass ratio = 2:1) at 50°C and 220 rpm for 72 h. After the reaction, the enzymatic reaction solution was heated in a boiling water bath for 10 min to inactivate the enzyme, then the enzymatic digest solution was obtained by vacuum filtration and monosaccharide composition and concentration was analyzed by using HPLC.

### 2.5 Extraction and analysis of medium-chain-length polyhydroxyalkanoate

The extraction and analysis of PHA were described previously (Poblete-Castro et al., 2013). Briefly, an appropriate amount of culture solution was taken and the cells were harvested by centrifugation at 4°C and 8,000 rpm for 10 min and washed with distilled water. The washed bacteria were then frozen at −80°C and lyophilized for 24 h. Then, the appropriate amounts of bacteria were weighed after drying, placed in a reactor containing 2 ml of esterification solution and 2 ml of chloroform, and esterified at 100°C for 4 h. The esterification solution was 3% sulfuric acid in methanol, and benzoic acid was added as an internal standard. After the esterification reaction was completed, pure water was added to the reaction mixture at room temperature, mixed evenly, and left standing for stratification. The lower organic phase was passed through a filter membrane to obtain the sample to be tested. The PHA was quantified by gas chromatography, with an injection volume of 1 µl. The starting temperature of the chromatographic separation column (Agilent HP-5) was 80°C and maintained for 1.5 min, followed by ramp to 140°C at 30°C/min. Then the temperature was increased to 240°C at 40°C/min and kept at 240°C for 4 min, and the entire program lasted 10 min. The PHA monomer was characterized by gas chromatography-mass spectrometry. The starting temperature of the chromatographic separation column (Agilent HP-FFAP) was 50°C and was maintained for 5 min. The temperature was then increased to 220°C at 5°C/min and kept for 20 min. The temperatures of inlet, ion source, and the interface were set as 220°C, 230°C, and 220°C, respectively.

### 2.6 Analytical methods

Cell optical density was measured at a wavelength of 600 nm (OD_600_) with UV-1200 spectrophotometer (Mapada, China). The OD_600_ value of two bacteria in the microbial consortium culture process was analyzed using the colony counting method. Based on the characteristics of kanamycin and gentamicin resistance in *E. coli* Δ4D (ACP-SCLAC), kanamycin and chloromycetin resistance in *P. putida* KTΔABZF (p2-a-J), the first step is to test the quantitative relationship between the number of colonies of the two bacteria and their OD_600_, respectively. Diluting the bacterial liquids with different OD_600_ to the appropriate multiples and spreading them on the corresponding resistant plates, the quantitative relationships between OD_600_ and the number of colonies of the two bacteria was obtained as: for *E. coli* Δ4D (ACP-SCLAC), 1OD_600_ = 3.25 × 10^7^ CFU/ml; for *P. putida* KTΔABZF (p2-a-J), 1OD_600_ = 1.80 × 10^6^ CFU/ml. Then, the co-culture solutions of different periods were diluted to a certain multiple and then coated on plates with kanamycin and gentamicin resistance, kanamycin and chloramphenicol resistance respectively. The OD_600_ of the two bacteria in the co-culture can be calculated then, based on the different characteristics of resistance in *E. coli* Δ4D (ACP-SCLAC) and *P. putida* KTΔABZF (p2-a-J), and the above quantitative relationships.

Acetic acid, glucose, and xylose were quantified in the culture supernatant using an Ultimate 3000 HPLC (Dionex, Sunnyvale, CA, United States) equipped with an Aminex HPX-87H ion-exchange column (Bio-Rad, United States) operating at 65°C and a differential refraction detector, with 5 mM H_2_SO_4_ as the mobile phase at a flow rate of 0.6 ml/min. Extraction and detection of FFAs were performed as described previously (Eiteman and Altman, 2006). Briefly, 2 ml of fermentation broth was centrifuged at 12,000 rpm for 5 min, and the supernatant was collected. Then, 200 μl of glacial acetic acid and 150 mg of internal standard (undecanoic acid) were added to the supernatant, mixed with 2 ml of extractant (n-hexane: chloroform = 4: 1 v/v), and shaken thoroughly. After standing still, the above-mentioned mixed solution was placed in the inner lining of the reactor, and allowed to stand overnight in a fume hood. After the organic reagents were completely volatilized, 1 ml of esterification solution (chloroform: methanol: sulfuric acid = 10: 8.5: 1.5, v/v) was added, the reactor was sealed and placed in an oven at 100°C for 1 h. After the reaction was completed, pure water was added to the reaction mixture at room temperature, mixed evenly, and left standing for stratification. The lower organic phase was passed through a filter membrane to obtain the sample to be tested. The FFAs were quantified by gas chromatography, with an injection volume of 1 µl. The starting temperature of the chromatographic separation column (Agilent HP-5) was 60°C and maintained for 3 min. Then the temperature was increased to 250°C at 10°C/min and kept at 250°C for 10 min, and the entire program lasted 30 min.

### 2.7 Statistical data analysis

All experiments in this study were performed in triplicate. Values were presented as the mean ± standard deviation for data. For multiple comparison, *p* values were derived from one-way ANOVA with Tukey’s multiple comparisons test using GraphPad Prism version 9.0.0 for Windows. For all comparisons, *p* < 0.05 was considered statistically significant. Graphs were prepared in Origin (version 2021b).

## 3 Results and discussion

### 3.1 Metabolic engineering of *P. putida* for increased medium-chain-length polyhydroxyalkanoate production

The design and construction of the artificial microbial consortium was based on the “nutrient supply-detoxification” inter-bacterial relationship with acetic acid and FFAs as intermediate metabolites. In this approach, the targeted enhancement of metabolic functions in different chassis cells can help enhance the capacity of product synthesis in the microbial consortium. Based on the metabolic pathway of *P. putida* KT2440 to synthesize mcl-PHA, we applied various metabolic engineering strategies with the goal of producing mcl-PHA from lignocellulosic hydrolysate in the artificial microbial consortium. These included: 1) elimination of mcl-PHA solubilization (Salvachua et al., 2020a), 2) weakening of the fatty acid β-oxidation pathway to reduce the flux of intermediates to FFAs degradation (Liu et al., 2011), 3) inhibition of competing pathways (Borrero-de Acuña et al., 2014), and 4) enhancement of the conversion of β-oxidation intermediates to synthesize mcl-PHA precursors (Zhang et al., 2021). To eliminate mcl-PHA depolymerization, the *phaZ* gene encoding PHA depolymerase was knocked out in the wild-type strain *P. putida* KT2440 as well as the engineered KTΔAB strain in which the *fadA* and *fadB* genes had been knocked out previously, resulting in the engineered strains KT2440ΔZ and KTΔABZ, respectively. In order to reduce the degradation of the β-oxidation intermediate 3-hydroxyacyl-CoA, the *yqeF* gene encoding acetyl coenzyme A acetyltransferase and the *paaJ* gene encoding β-ketohexanoyl coenzyme A thiolase were knocked out in KTΔABZ to produce the engineered strains KTΔABZF and KTΔABZFJ. In addition, the *tctA* gene encoding a tricarboxylic acid transport protein was knocked out in strain KTΔABZFJ to produce KTΔABZFJT, which can be used to disrupt the tricarboxylic acid transport system aiming to divert energy toward PHA accumulation. To further increase the conversion efficiency of acetic acid and FFAs in these knockout strains, thus contributing to the availability of mcl-PHA precursors, the *acs* gene encoding acetyl coenzyme A synthase and the *phaJ* gene encoding enoyl coenzyme A hydratase were overexpressed to produce the strains KTΔABZF (p2-a-J) and KTΔABZFJ (p2-a-J). To enhance the polymerization of the PHA precursor 3-hydroxyacyl-CoA to mcl-PHA, the *phaC1* and *phaC2* genes encoding PHA polymerase (Li et al., 2021) were expressed using the T3 promoter or Tac promoter, to produce strains KTΔABZF (p2_T3_-C1C2) and KTΔABZF (p2_Tac_-C1C2), respectively. Furthermore, the *acs* and *phaJ* genes were overexpressed using the T3 promoter, while the *phaC1* and *phaC2* genes were overexpressed using the Tac promoter, resulting in the strain KTΔABZF (p2-a-J-C1C2). In order to overcome the inhibitory effect of lignin in the lignocellulosic hydrolysate, the *SCLAC* gene encoding laccase derived from *S. azureus* was expressed heterologously using the T3 promoter or Tac promoter, resulting in the strains KTΔABZF (p2_T3_-SCLAC) and KTΔABZF (p2_Tac_-SCLAC), respectively.

#### 3.1.1 Engineering the endogenous metabolism of *P. putida* to increase the production of medium-chain-length polyhydroxyalkanoate

**FIGURE 2:**
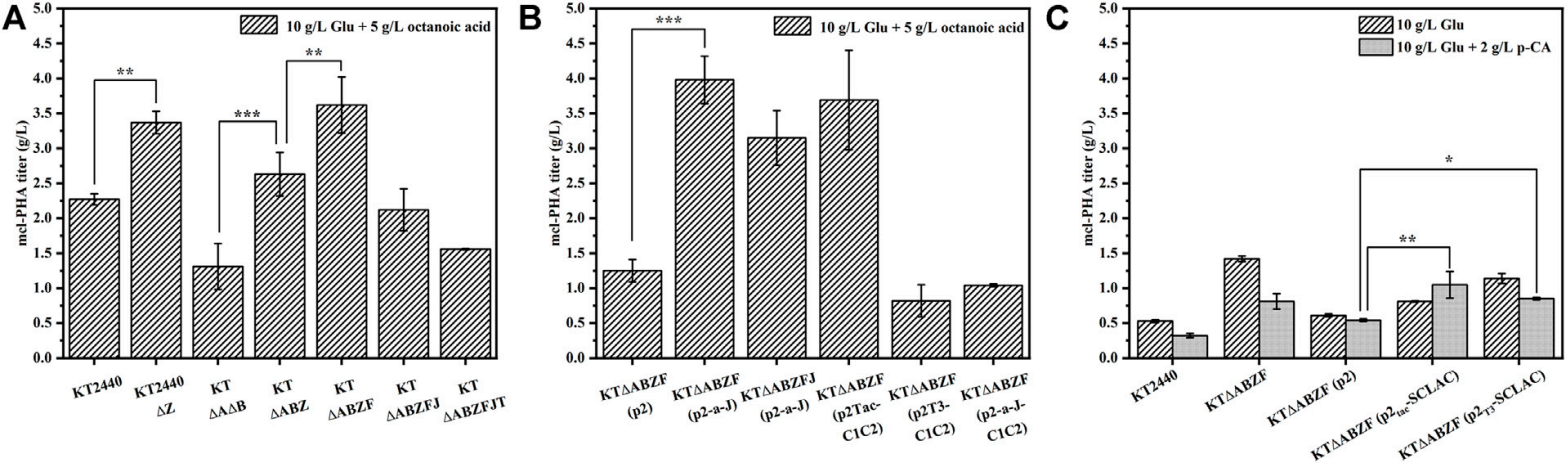
The ability of the engineered *P. putida* by genetic modifications to produce mcl-PHA. **(A) **The engineered *P. putida* by knocking out corresponding genes. **(B)** The engineered *P. putida* by overexpression corresponding genes. **(C)** The engineered *P. putida* by heterologous expression of laccase. The error bars indicate the standard deviation of triplicate experiments. *p < 0.05; **p < 0.01; ***p < 0.001.

To test the mcl-PHA production capacity of the different metabolically engineered *P. putida* strains introduced above, we used 10 g/L glucose and 5 g/L octanoic acid as substrates for the synthesis of mcl-PHA in a two-step fermentation lasting 60 h. After the engineered *P. putida* as incubated with glucose for 24 h, octanoic acid was added to the medium as a precursor of mcl-PHA. The wild-type strain KT2440 and the starting engineered strain KTΔAB were included as controls. As shown in Figure 2A, the intracellular mcl-PHA content of all the engineered strains increased to some extent compared to the wild-type strain KT2440, as well as the initial engineered strain KTΔAB. As expected, the intracellular content of mcl-PHA in the engineered KT2440ΔZ and KTΔABZ with knockout of the *phaZ* gene were enhanced compared to their respective parental strains. This led to a significant increase of PHA production, which was consistent with the literature (Salvachúa et al., 2020b). This suggests that knocking out the *phaZ* gene encoding PHA depolymerase to eliminate PHA depletion is an effective strategy to increase PHA production. The mcl-PHA content of the KT∆ABZF strain with the *yqeF* gene knocked based on KT∆ABZ reached 3.62 g/L, which was 1.59 times higher than that of the original strain KT2440 and 2.76 times higher than that of the initial engineered strain KT∆AB. Acetyl coenzyme A acetyltransferase is encoded by the *yqeF* gene (Zhuang and Qi, 2019), and the deletion of this gene resulted in a large accumulation of acetyl-CoA, weakening the fatty acid β-oxidation cycle. Further knockout of the *paaJ* gene resulted in a strong blockage of the fatty acid β-oxidation cycle, as shown in Supplementary Table S3, and although a high intracellular content of PHA was accumulated (85.89 wt%), the strain’s growth was significantly inhibited because the carbon flux from the weakened fatty acid β-oxidation was mainly redirected toward the synthesis of PHA, which reduced the intracellular energy supply, resulting in poor biomass accumulation of the engineered strain KT∆ABZFJ, as evidenced by a significant decrease of cell dry weight (CDW). By contrast, KT∆ABZFJT with a further knock out of the *tctA* gene encoding the tricarboxylic acid transport protein based on KT∆ABZFJ, did not perform as well as reported for a similar strain in a previous study (Tao et al., 2017), probably because the simultaneous blockage of the tricarboxylic acid cycle and severe inhibition of the fatty acid β-oxidation cycle resulted in the absence of reducing coenzymes essential for cell growth, which could not complete oxidative phosphorylation to provide the necessary energy for cell growth (Zhuang et al., 2014; Tao et al., 2017).

The mcl-PHA production capacity of the engineered strains was characterized under the same conditions. To exclude the effect of the introduced plasmid pBBR1MCS2 on the engineered bacteria, the KT∆ABZF (p2) strain containing the empty vector was used as a control. The content of intracellular PHA in engineered KT∆ABZF (p2-a-J) overexpressing the *acs* and *phaJ* genes reached 91.96 wt% (Supplementary Table S3) and the mcl-PHA titer was 3.98 g/L (Figure 2B), which was 3.18 times higher than that of the control strain KT∆ABZF (p2). However, similar co-expression of both *phaJ* and *fabG* in *P. putida* KCTC1639 did not yield satisfactory results in a previous study (Vo et al., 2008). Nevertheless, the cell growth and mcl-PHA accumulation of the overexpression strain KT∆ABZFJ (p2-a-J) were improved compared with its parental strain KT∆ABZFJ, indicating that overexpression of *acs* could promote the production of acetyl coenzyme A, thus alleviating the cell growth limitation of KT∆ABZFJ due to the severe weakening of fatty acid β-oxidation. At the same time, the *phaJ* gene enhanced the conversion efficiency of FFAs into mcl-PHA precursors *via* the β-oxidation pathway. In addition, overexpression of *phaC1* and *phaC2* using different promoters resulted in significant differences of cell growth and mcl-PHA accumulation in the engineered bacteria (Supplementary Table S3). Accordingly, the intracellular content of mcl-PHA reached 96.77 wt% in KT∆ABZF (p2_Tac_-C1C2) using the Tac promoter, while the growth of KT∆ABZF (p2_T3_-C1C2) using the T3 promoter was significantly inhibited. This might be due to the fact that T3-mediated expression did not achieve the expected effect of polymerizing PHA precursors and the strain could not effectively utilize FFAs to accumulate mcl-PHA, while still incurring a metabolic burden due to the overexpression vector. Additionally, the mcl-PHA production of the co-overexpression strain KT∆ABZF (p2-a-J-C1C2) was not further enhanced, probably due to the increased metabolic burden. The monomer fractions of PHA accumulated by these metabolically engineered strains are summarized in Supplementary Table S3. The PHA contained all even medium-chain-length monomers from C6-C14, whereby 3-hydroxyoctanoate (3HO) accounted for the largest fraction. For example, strain KT∆ABZF (p2-a-J) accumulated 86.69 mol% of 3HO. As expected, the utilization of octanoic acid as precursor caused the accumulation of C8 monomers due to the disruption of FFAs degradation.

#### 3.1.2 Engineering *P. putida* by heterologous expression of laccase to increase inhibitor tolerance

Since this study aimed to produce mcl-PHA from lignocellulosic hydrolysate, it was necessary to decrease the potential inhibitory effect of lignin on the engineered *P. putida*. Accordingly, the *SCLAC* gene encoding laccase from *S. azureus* was introduced into the engineered strain KT∆ABZF and heterologously expressed using the T3 and Tac promoters, respectively. To examine the effect of heterologous expression of laccase on the accumulation of mcl-PHA by the engineered *P. putida*, the engineered bacteria were incubated in M9 medium with a final glucose concentration of 10 g/L for 24 h, followed by the addition of 2 g/L of the lignin-derived monomer p-coumaric acid (p-CA) as a substrate until 60 h (Figure 2C). The medium with only 10 g/L glucose was used for comparison, while the KT2440, KT∆ABZF and KT∆ABZF (p2) strains were included as controls. In all engineered strains, the CDW was significantly lower in the medium with added p-CA compared to glucose only (Supplementary Table S3), indicating that the biomass accumulation was significantly inhibited, which was caused by the cytotoxicity of p-CA itself (Sarlaslani, 2007). In contrast to the CDW, the intracellular content of PHA was increased in all the engineered strains in the medium supplemented with p-CA, which was attributed to the fact that *P. putida* KT2440 has a native ability to break down aromatic compounds, and convert p-CA into mcl-PHA *via* protocatechuic acid and the β-KA pathway to complete the lignin depolymerization and synthesis of FFAs (Linger et al., 2014). In particular, KT∆ABZF (p2_T3_-SCLAC) and KT∆ABZF (p2_Tac_-SCLAC) both showed a significant increase in the intracellular content of mcl-PHA, with KT∆ABZF (p2_Tac_-SCLAC) accumulating close to 50 wt% mcl-PHA. At the same time, the mcl-PHA titer of both engineered strains was increased compared to KT∆ABZF (p2), especially when p-CA was added as a substrate, whereby the PHA titer of KT∆ABZF (p2_Tac_-SCLAC) was higher (1.0 g/L) than that of the corresponding strain with only glucose as substrate. This indirectly indicates a positive effect of heterologous expression of laccase on the potential utilization of lignocellulosic hydrolysate to produce mcl-PHA by the engineered *P. putida*. Unlike the previous use of octanoic acid as a precursor, the engineered strain utilizing only glucose accumulated PHA with predominantly 3-hydroxydecanoate (3HD) monomers, similar to the engineered bacteria in the medium supplemented with p-CA, with KT∆ABZF (p2_Tac_-SCLAC) accumulating the highest 3HD fraction of 56.75 mol% (Supplementary Table S3).

#### 3.1.3 Production of medium-chain-length polyhydroxyalkanoate by the engineered *P. putida* using mixed substrates

**FIGURE 3:**
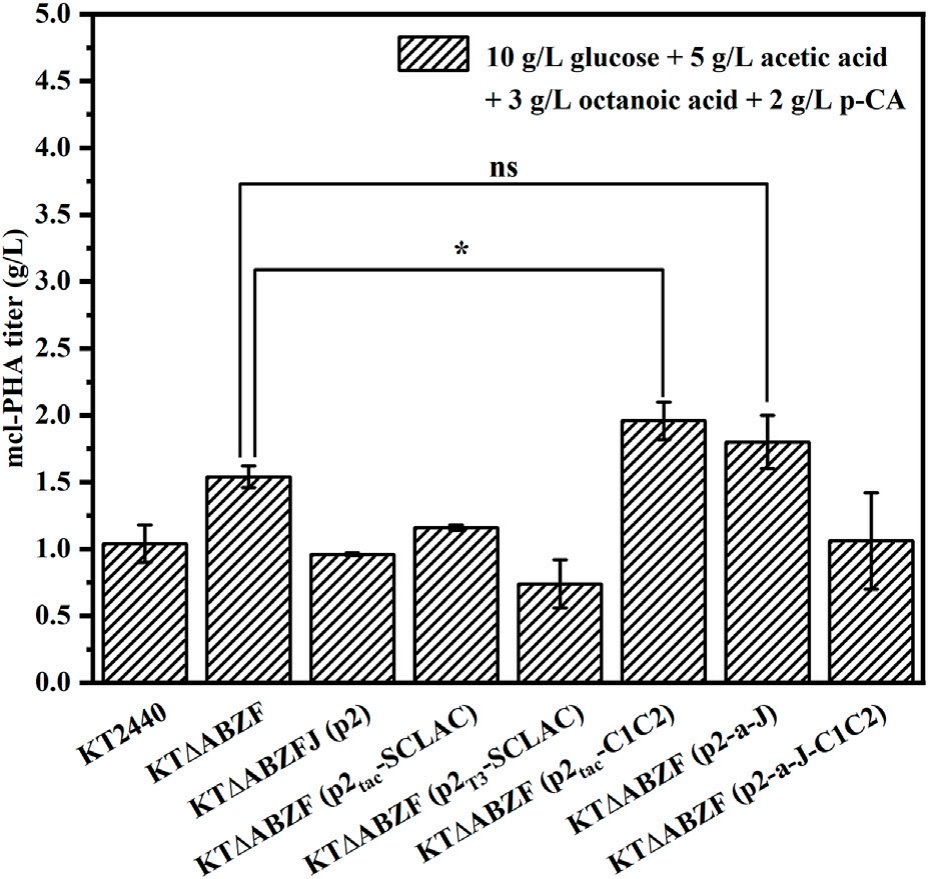
The performance of mcl-PHA production by the engineered *P. putida* using 10 g/L glucose, 5 g/L acetic acid, 3 g/L octanoic acid and 2 g/L p-CA as mixed substrates. The engineered *P. putida* were incubated with 10 g/L glucose, 5 g/L acetic acid, 3 g/L octanoic acid and 2 g/L p-CA as mix substrates for 60 h. The error bars indicate the standard deviation of triplicate experiments. *p < 0.05; **p < 0.01; ***p < 0.001; ns, no significance.

When the artificial microbial consortium produces mcl-PHA from lignocellulosic hydrolysate, the potential carbon sources utilized by engineered *P. putida* include glucose, acetic acid, FFAs and aromatic compounds. In order to test the production of mcl-PHA by engineered *P. putida* using mixed substrates, we fermented the engineered *P. putida* in medium containing 10 g/L glucose, 5 g/L acetic acid, 3 g/L octanoic acid and 2 g/L p-CA as mixed substrates for 60 h. The original strain KT2440, the parental strain KT∆ABZF and the engineered strain KT∆ABZF (p2) containing the empty vector pBBR1MCS-2 were also fermented under the same conditions as controls. As shown in Figure 3, the engineered strains KT∆ABZF (p2_Tac_-C1C2) and KT∆ABZF (p2-a-J) showed superior performance in terms of intracellular content and final titer of mcl-PHA. These two engineered strains accumulated comparable amounts of mcl-PHA, with similar titers of 1.96 and 1.80 g/L, respectively. The remaining engineered strains showed a decrease of CDW, intracellular PHA content (Supplementary Table S3) and final PHA titer compared to KT∆ABZF, indicating that acetic acid and p-CA may also decrease cell growth (Sarlaslani, 2007; Ludwig et al., 2013) in addition to the effect of the vector itself. In the mcl-PHA synthesized by all strains, 3HO still accounted for the highest percentage of monomers, indicating that engineered *P. putida* could efficiently convert octanoic acid for the accumulation of mcl-PHA.

In summary, the results showed that knockout of the *phaZ* gene encoding PHA depolymerase and moderate weakening of the fatty acid β-oxidation pathway could effectively improve the mcl-PHA production, where by the product titer of the engineered strain KT∆ABZF reached 3.62 g/L using 10 g/L glucose and 5 g/L octanoic acid as substrates, which was 2.76 times higher than that of the initial engineered strain KT∆AB. The engineered strain KT∆ABZF (p2-a-J), with an additionally enhanced acetic acid assimilation pathway and improved conversion of fatty acid β-oxidation intermediates, exhibited a further increase of the mcl-PHA titer to 3.98 g/L, which was 3.04 times higher than that of KT∆AB. In addition, heterologous expression of laccase also had a positive effect on the ability of the engineered *P. putida* to use the lignin derivative p-CA for mcl-PHA production, whereby the engineered strain KT∆ABZF (p2_Tac_-SCLAC), which heterologously expressed *SCLAC* using the Tac promoter, accumulated 49.39% of mcl-PHA, which was 142.82% higher than in the wild-type strain KT2440. Finally, we tested the mcl-PHA production capacity of the engineered bacteria in lignocellulosic hydrolysate using simulated mixed substrates, and the results showed that the engineered strain KT∆ABZF (p2-a-J) produced 1.80 g/L mcl-PHA, which was 73.08% higher compared to the wild-type strain KT2440.

### 3.2 Metabolic engineering of *E. coli* for enhanced substrate supply for the engineered *P. putida* to produce medium-chain-length polyhydroxyalkanoate

Based on our previous studies, engineered *E. coli* has been shown to preferentially utilize xylose in media containing both glucose and xylose, while slowly metabolizing glucose and secreting the intermediate metabolites acetate and FFAs (Liu et al., 2020; Zhu et al., 2021). In this study, we showed that engineered *P. putida* can use FFAs as a related carbon source to increase the supply of precursors for mcl-PHA production and improve the final titer. In addition, it has been shown that overexpression of relevant genes in the FFAs metabolic pathway of *E. coli* is an effective strategy to improve FFAs production (Lennen et al., 2010; Zhang et al., 2012). Therefore, increasing the FFAs and acetic acid production of *E. coli* is essential for efficient synthesis of mcl-PHA from lignocellulosic hydrolysates using artificial microbial consortia. In this sturdy, we used *E. coli* ∆4D as the starting strain and overexpressed the *tesA* gene (Steen et al., 2010) encoding thioesterase, the *fabZ* gene (Yu et al., 2011) encoding hydroxylated acyl-ACP dehydratase and the *fabD* gene (Zhang et al., 2012) encoding acyltransferase to produce the engineered *E. coli* ∆4D (*tesA*), *E. coli* ∆4D (*fabZ*) and *E. coli* ∆4D (*fabD*), respectively. Among them, the enzyme encoded by the *tesA* gene catalyzes the last step of FFAs synthesis and can release free FFAs from acyl ACP, while the enzymes encoded by the *fabZ* and *fabD* genes enhance the synthesis of acyl ACP. To further enhance the supply of precursors for FFAs synthesis, the thioesterase gene of the ricin acyl carrier protein (Hernández Lozada et al., 2018) was co-expressed with the above three genes in different combinations to produce the engineered strains *E. coli* ∆4D (AD), *E. coli* ∆4D (AAD), *E. coli* ∆4D (AZD) and *E. coli* ∆4D (AAZD), respectively. In addition, to achieve the secretion of FFAs and acetic acid by engineered *E. coli* grown on lignocellulosic hydrolysate, we added another vector containing the *SCLAC* gene to *E. coli* ∆4D (ACP) and constructed the engineered *E. coli* ∆4D (ACP-SCLAC).

#### 3.2.1 Engineering *E. coli* to produce acetic acid and free fatty acids from xylose

**FIGURE 4:**
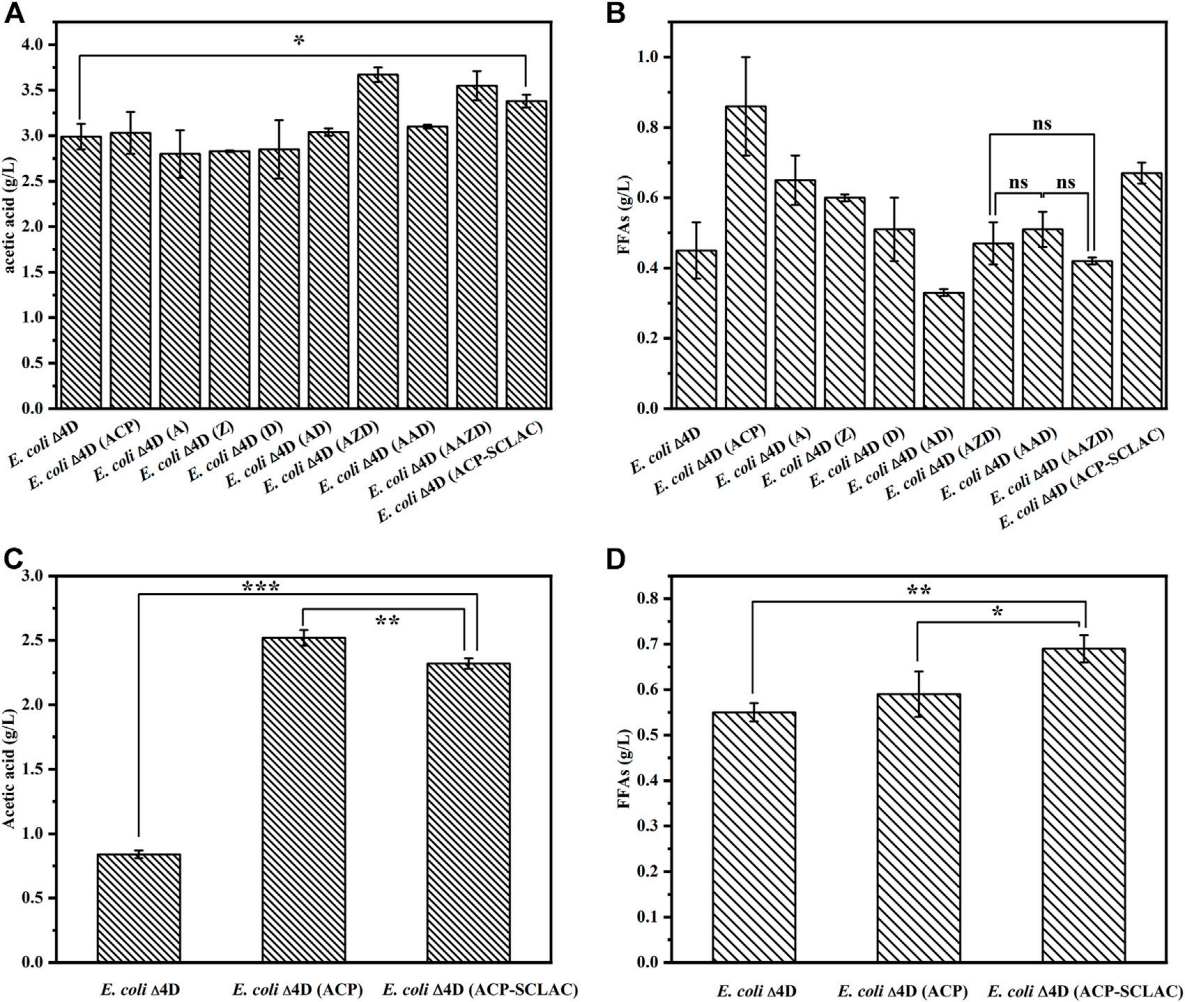
The production of acetic acid and FFAs by the engineered *E. coli* using different substrates. **(A)** The production of acetic acid by engineered *E. coli* using 10 g/L xylose as substrates for 64 h. **(B)** The production of FFAs by engineered *E. coli* using 10 g/L xylose as substrates for 64 h. **(C)** The production of acetic acid by engineered *E. coli* using lignocellulosic hydrolysate as substrates for 64 h. **(D)** The production of FFAs by engineered *E. coli* using lignocellulosic hydrolysate as substrates for 64 h. The error bars indicate the standard deviation of triplicate experiments. *p < 0.05; **p < 0.01; ***p < 0.001; ns, no significance.

The ability of the engineered *E. coli* strains to produce acetic acid (Figure 4A) and FFAs (Figure 4B) was assessed in shake-flask fermentations with 10 g/L xylose as substrate for 64 h. The starting strains *E. coli* ∆4D and *E. coli* ∆4D (ACP) were included as controls. As shown in Figure 4A, the engineered strains that individually overexpressed the *tesA*, *fabZ* and *fabD* genes in the fatty acid synthesis pathway, did not have an enhanced ability to produce acetic acid, whereas the engineered strains that overexpressed these genes in different combinations produced more acetic acid. Specifically, the engineered *E. coli* ∆4D (AZD) and *E. coli* ∆4D (AAZD) respectively produced 3.67 and 3.55 g/L acetic acid, representing 22.74 and 18.73% increases compared to *E. coli* ∆4D. By contrast, the production of FFAs was increased in the engineered strains with individual overexpression of *tesA*, *fabZ* and *fabD*, as shown in Figure 4B. However, the FFAs production of *E. coli* ∆4D (A) overexpressing the *tesA* gene was reduced to 0.65 g/L, compared to 0.86 g/L produced by the engineered *E. coli* ∆4D (ACP). This indicated that the overexpression of the gene encoding thioesterase had a significant effect on FFA secretion in engineered *E. coli*, in agreement with the literature (Lee et al., 2013). Compared to the control strain *E. coli* ∆4D, the acetate and FFAs production of *E. coli* ∆4D (A) increased by 91.11% and 44.44%, respectively, indicating that overexpression of the *tesA* gene had a positive effect. However, no significant increase in FFAs production was observed in the co-overexpression strain compared to *E. coli* ∆4D. In terms of metabolic pathways, co-overexpression may have led to excessive flux through the fatty acid synthesis pathway, which may have resulted in the depletion of the acetyl-CoA pool in the engineered *E. coli* (Lu et al., 2008), thus leading to reduced xylose utilization (Supplementary Figure S1). In addition, the dual-vector strain *E. coli* ∆4D (ACP-SCLAC) secreted up to 3.38 g/L acetic acid and 0.67 g/L FFAs. The FFAs production capacity of *E. coli* ∆4D (ACP-SCLAC) was reduced compared to *E. coli* ∆4D (ACP), which was ascribed to the increased metabolic burden from the dual plasmid vector. By contrast, both acetic acid and FFAs production were significantly higher compared to *E. coli* ∆4D, and no residual xylose was detected in the medium after 64 h (Supplementary Figure S1). Therefore, establishing a balance between product accumulation and strain growth is necessary to increase the production of acetic acid and FFAs in engineered *E. coli*.

#### 3.2.2 Engineering *E. coli* to utilize lignocellulosic hydrolysate for medium-chain-length polyhydroxyalkanoate production

In view of the above results on the production of acetic acid and FFAs by engineered *E. coli* using xylose, as well as the design of the reconstructed artificial microbial consortium for the production of mcl-PHA using lignocellulosic hydrolysate, we selected the three engineered strains *E. coli* ∆4D, *E. coli* ∆4D (ACP) and *E. coli* ∆4D (ACP-SCLAC) for fermentation experiments using lignocellulosic hydrolysate obtained through acid pretreatment of corn straw. The lignocellulosic hydrolysate used in this study contained 1.63 g/L glucose, 21.39 g/L xylose and 3.13 g/L arabinose, which was in agreement with a previous study using a similar pretreatment method (Avci et al., 2013). The lignocellulosic hydrolysate and M9 medium were mixed at a 9:1 (v/v) ratio and used as the culture medium (Salvachua et al., 2015). As shown in Figures 4C,D, the acetic acid titer of the engineered *E. coli* ∆4D (ACP-SCLAC) reached 2.32 g/L, which was 2.76 times higher than that of the starting strain *E. coli* ∆4D. Although the increase in FFAs production of *E. coli* ∆4D (ACP-SCLAC) compared to *E. coli* ∆4D (ACP) was not significant, the heterologous expression of laccase enhanced the ability of the engineered *E. coli* ∆4D (ACP-SCLAC) to use lignocellulosic hydrolysate to some extent. In particular, the engineered *E. coli* ∆4D (ACP-SCLAC) secreted 0.69 g/L FFAs, which was close to the titer obtained on xylose. Combined with the changes in the concentration of major sugars in the medium of the lignocellulosic hydrolysate (Supplementary Table S4), it was found that engineered *E. coli* did not significantly deplete the arabinose that was also available in the medium, while the initial small amount of glucose in the medium was completely consumed, along with almost 5 g/L of xylose. Theoretically, *E. coli* can use glucose, xylose as well as arabinose, and although the strains used in this study were genetically engineered to prefer xylose and slowly metabolize glucose, carbon catabolite repression (CCR) was not completely released. Similar studies have shown that this appears to be due to residual activity of the glucose PTS (phosphotransferase system) (Xiao et al., 2011). In conclusion, the engineered *E. coli* ∆4D (ACP-SCLAC) exhibited improved acetic acid and FFA production capacity using xylose or lignocellulosic hydrolysate as substrate and was selected as one of the functional strains to construct the artificial microbial consortium.

### 3.3 Reconstruction and optimization of the artificial microbial consortium using mixed sugars

Artificial microbial consortia are a novel and promising platform for the biosynthesis of target products that can distribute the metabolic burden among individual strains (Li et al., 2019; Roell et al., 2019). In this study, the “nutrient supply-detoxification” principle was used to reconstruct a synthetic microbial consortium consisting of two engineered strains belonging to different species (Figure 1). First, the best-performing engineered *E. coli* ∆4D (ACP-SCLAC) was selected as the nutrient supply module for the microbial consortium, while the most promising strain for mcl-PHA production, *P. putida* KT∆ABZF (p2-a-J), was selected to perform the task of utilizing the complex components of lignocellulosic hydrolysate while detoxifying acetic acid and FFAs for increased productivity.

**FIGURE 5:**
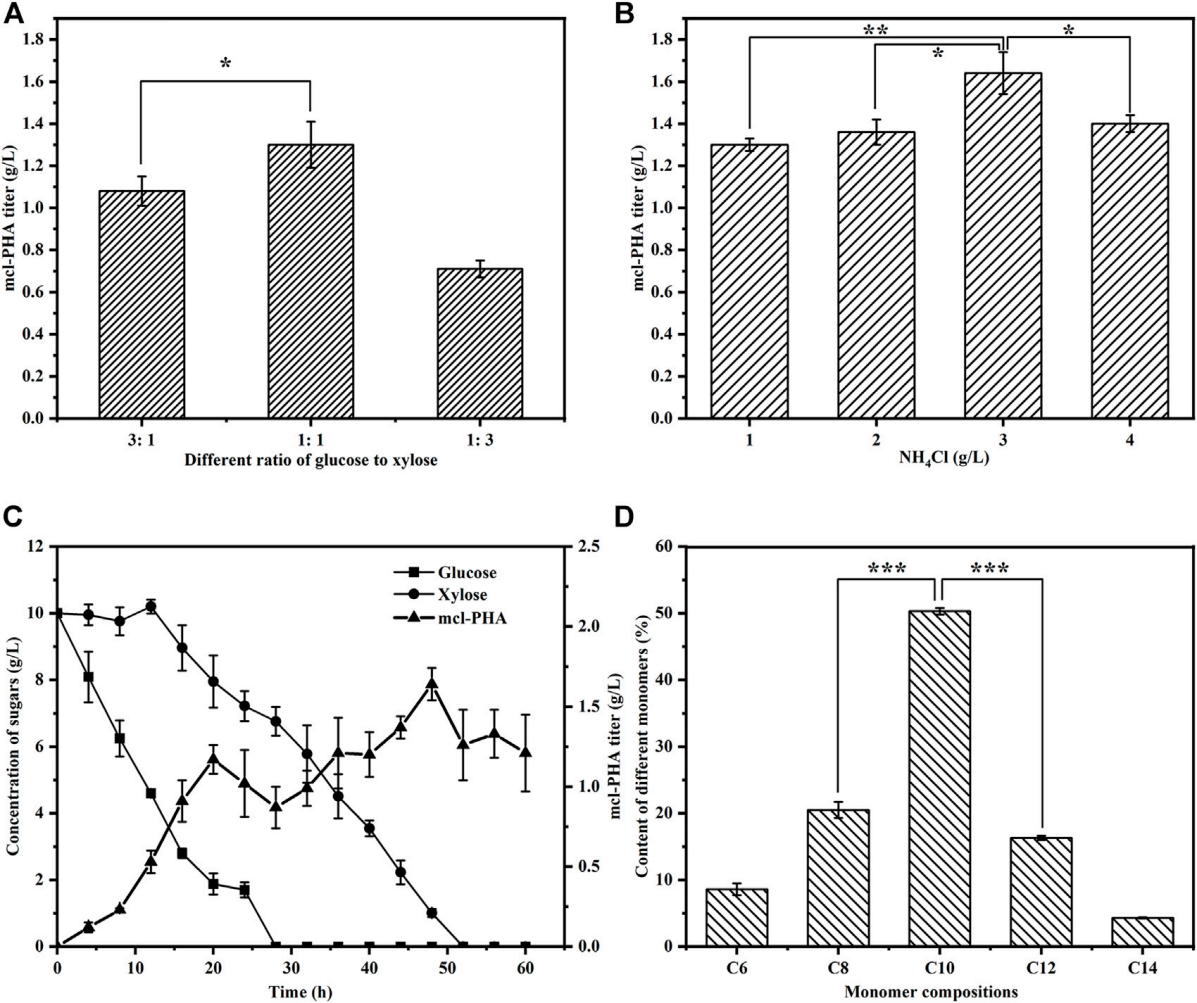
The production of mcl-PHA by the *P. putida*-*E. coli* microbial consortium using M9 medium with added glucose and xylose. **(A)** The mcl-PHA titer in the microbial consortium with different mixed sugar ratios and the total concentrations of mix sugars were 20 g/L. **(B)** The mcl-PHA titer in the microbial consortium at different nitrogen concentration. **(C)** The residual contents of different sugars by the microbial consortium. **(D)** The monomer composition of mcl-PHA produced by the microbial consortium. The error bars indicate the standard deviation of triplicate experiments. *p < 0.05; **p < 0.01; ***p < 0.001; ns, no significance.

One of our goals in designing the artificial microbial consortium was to use lignocellulosic hydrolysate as an inexpensive substrate for the production of mcl-PHA. We therefore first tested the ability of the artificial microbial consortium to produce mcl-PHA using glucose and xylose, the main monosaccharides found in lignocellulosic hydrolysate, as mixed carbon sources. The construction of an artificial microbial consortium that can fully coordinate the interactions and synergistic growth of bacteria is the key to optimizing the co-culture strategy (Kong et al., 2018). Our previous studies have demonstrated that the inoculation ratio of consortia has a small effect on the titer of mcl-PHA, while the inoculation order between engineered bacteria has a decisive effect on strain dominance in the co-culture (Zhu et al., 2021), which in turn affects the titer of mcl-PHA. We therefore added *E. coli* ∆4D (ACP-SCLAC) and *P. putida* KT∆ABZF (p2-a-J) at a 1:2 ratio, whereby the engineered *P. putida* was added to the medium 12 h before the engineered *E. coli* ∆4D (ACP-SCLAC). Moreover, due to the preference of the two species for glucose and xylose, as well as the significant effect of the intermediate metabolites (acetic acid and FFA) on mcl-PHA synthesis, adjusting the ratio of glucose and xylose in the mixed sugar medium is likely to affect the final titer of mcl-PHA. Consequently, we tested three different ratios of mixed sugar substrates to test the ability of the artificial microbial consortium to produce mcl-PHAs under nutrient-limited conditions with 1 g/L NH_4_Cl. As shown in Figure 5A, the amount of mcl-PHA accumulated in the medium was similar at glucose-xylose ratios of 1:1 and 3:1, with the highest titer reaching 1.30 g/L in the former. By contrast, only 0.71 g/L mcl-PHA was detected in the medium with a glucose-xylose ratio of 1:3. This was because the engineered *P. putida* KT∆ABZF (p2-a-J) first used glucose to rapidly accumulate biomass, after which the engineered *E. coli* ∆4D (ACP-SCLAC) subsequently supplied acetic acid and FFAs to the engineered *P. putida* KT∆ABZF (p2-a-J) to produce mcl-PHA. For the engineered *P. putida* KT∆ABZF (p2-a-J), which was inoculated first, the carbon source available in the medium consisting of glucose and xylose (1:3) was inadequate to accumulate sufficient biomass, which was positively correlated with the synthesis of mcl-PHA, so that the biomass deficiency affected the subsequent mcl-PHA synthesis. This is also evidenced by the utilization of mixed sugars in the artificial microbial consortium (Supplementary Figure S2).

However, under nitrogen-limited conditions, the post-inoculated *E. coli* ∆4D (ACP-SCLAC) did not seem to be affected by the CCR and it could continuously consume xylose (Supplementary Figure S2), but the overall xylose utilization rate was low and there was still a small amount of residual xylose in the medium at the end of fermentation. This indicates that the nitrogen-limited conditions affected xylose utilization by *E. coli* ∆4D (ACP-SCLAC) in the co-culture system. In order to further improve the utilization of xylose by the engineered *E. coli*, the nitrogen source concentration had to be increased, but nitrogen source limitation promotes the accumulation of mcl-PHA, so exploring the appropriate nitrogen source concentration to achieve a balance between the two engineered bacteria is crucial for the production of mcl-PHA (Sangani et al., 2019). We therefore increased the concentration of the nitrogen source (NH_4_Cl) to 3 g/L, while using glucose and xylose at a 1:1 ratio as the carbon source. As shown in Figure 5C, glucose was rapidly depleted as before, but the increase of nitrogen source caused xylose to be depleted at 52 h of fermentation. In addition, the two-species consortium accumulated more mcl-PHA under these conditions. As shown in Figure 5B, the mcl-PHA titer reached 1.64 g/L, which was 26.15% higher than before. Similar to mcl-PHA produced by engineered *P. putida* in pure culture in a previous study (Yang et al., 2019), the mcl-PHA produced by the consortium in this study mainly contained medium-length monomers, with C10 as the most abundant monomer (Figure 5D), accounting for 50.30% of the total mcl-PHA content. This phenomenon was previously shown to be related to the fact that the main substrate utilized by the engineered *P. putida* was glucose (Liu et al., 2020).

### 3.4 Production of medium-chain-length polyhydroxyalkanoate from lignocellulosic hydrolysate using the artificial microbial consortium

**FIGURE 6:**
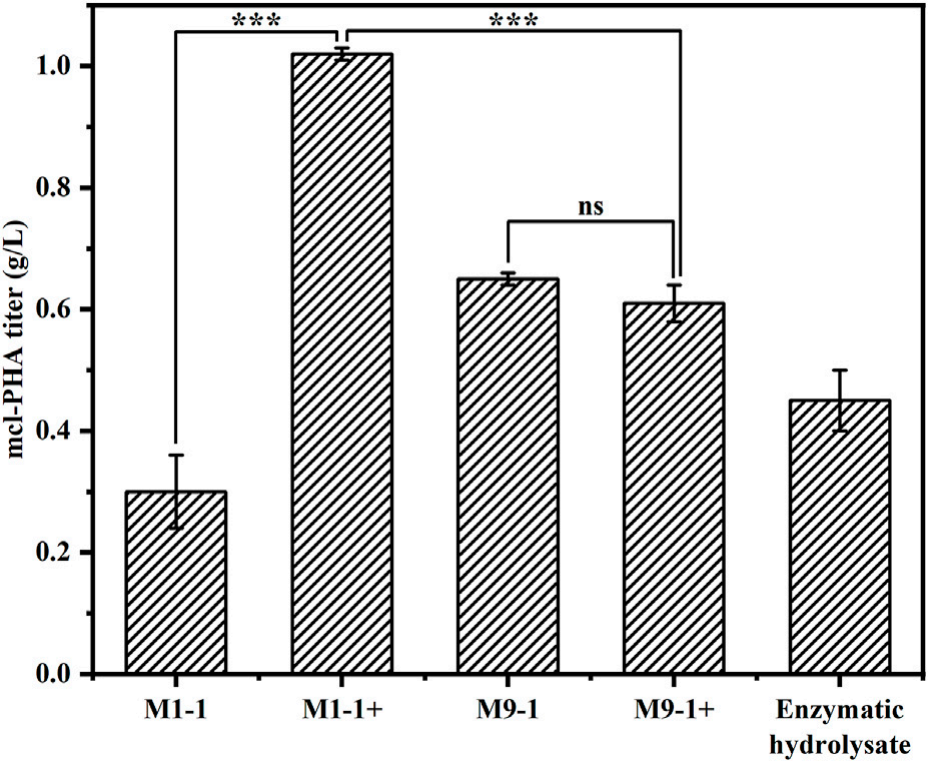
The production of mcl-PHA using lignocellulosic biomass by the *P. putida*-*E. coli* microbial consortium. M1-1: The ratio of acid-pretreatment solution of corn straw and M9 medium was 1:1; M1-1+: The ratio of acid-pretreatment solution of corn straw and M9 medium was 1:1 and 10 g/L glucose was added. M9-1: The ratio of acid-pretreatment solution of corn straw and M9 medium was 9:1; M9-1+: The ratio of acid-pretreatment solution of corn straw and M9 medium was 9:1 and 10 g/L glucose was added.

To explore the productivity of the consortium in the actual lignocellulosic hydrolysate, we used corn straw as raw material and pretreated it with acid to obtain a pretreatment solution rich in xylose and a small amount of glucose (21.39 g/L xylose, 1.63 g/L glucose, and 3.13 g/L arabinose). The straw solids that remained after acid pretreatment were further treated with cellulase and hemicellulose degrading enzymes, resulting in an enzymatic hydrolysate contained mainly 9.29 g/L glucose and 8.59 g/L xylose according to HPLC. The ability of the artificial microbial consortium to produce mcl-PHA in shake flasks was tested using the above acid pretreatment solution and enzymatic digest as substrates, respectively. As shown in Figure 6, the consortium performed best in the medium based on the pretreatment solution with added glucose (M1-1+), accumulating 1.02 g/L mcl-PHA. However, the addition of glucose seemed to have no significant effect on the synthesis of mcl-PHA by the consortium using a mixture of pretreatment solution and M9 medium at a ratio of 9:1, resulting in similar mcl-PHA production. Specifically, in the pretreatment solution medium without glucose addition (M9-1), the consortium accumulated 0.65 g/L mcl-PHA. Although the initial glucose concentration in the medium was only 2.03 g/L, the engineered *P. putida* KT∆ABZF (p2-a-J) which was seeded first nevertheless accumulated a certain amount of biomass, and subsequently used the intermediate metabolites acetic acid and FFAs for growth and mcl-PHA accumulation. By contrast, the consortium only accumulated 0.30 g/L mcl-PHA in the pretreatment medium without the addition of glucose (M1-1), which was mainly attributed to insufficient initial biomass. As shown in Supplementary Table S5, the two-species consortium was less efficient in utilizing sugars from the enzymatic hydrolysate compared to the acid pretreatment, with residual glucose and xylose still remaining at the end of fermentation. The decrease of substrate utilization in turn affected the product titer, and the consortium accumulated 0.45 g/L of mcl-PHA, presumably due to the lack of essential elements for microbial growth in the enzymatic hydrolysate. In addition, the high concentration of citrate contained in the hydrolysate may also have an inhibitory effect on microbial growth. At the same time, the chemicals from the pretreatment process have a strong inhibitory effect on the enzymatic process (Qin et al., 2016). However, the monomer composition of mcl-PHA was dominated by C8 and C10 (Table 1), while most recent studies using lignocellulose as a substrate produced scl-PHA (Linger et al., 2014; Liu et al., 2017; Arreola-Vargas et al., 2021).

**TABLE 1:** The monomer compositions of mcl-PHA produced by the microbial consortium using from lignocellulosic hydrolysate.

Strains	Monomer compositions (%)				
	3HHX (C6)	3HO (C8)	3HD (C10)	3HDD (C12)	3HTD (C14)
M1-1	7.61 ± 0.19	52.35 ± 1.14	20.54 ± 0.38	13.44 ± 0.66	6.06 ± 0.08
M1-1+	27.50 ± 1.69	28.25 ± 2.46	24.71 ± 2.58	12.81 ± 1.18	6.73 ± 0.39
M9-1	2.47 ± 0.44	66.51 ± 0.97	16.73 ± 1.76	10.83 ± 0.40	3.46 ± 0.82
M9-1+	16.33 ± 0.02	34.69 ± 1.40	22.04 ± 1.34	18.56 ± 1.48	8.38 ± 1.40
Enzymatic digest solution	8.15 ± 0.53	15.27 ± 1.21	57.83 ± 0.06	11.74 ± 0.51	7.00 ± 0.12

The main difference between the consortium reported in this manuscript and the previous studies is the target substrates. The main objective of our previous studies were to design and validate the ability of the microbial consortia to produce mcl-PHA from mixed sugars (Liu et al., 2020; Zhu et al., 2021), whose results demonstrated the good potential of these consortia, of which one could also produce mcl-PHA from lignocellulosic hydrolysate (0.434 g/L) (Liu et al., 2020). Nevertheless, the engineered *E. coli* ∆4D (ACP-SCLAC) in this study introduced heterologously expressed laccase aimed at increasing the tolerance and utilization of lignocellulosic hydrolysate by the consortium, while other genetic modifications were also made to increase the precursor supply of mcl-PHA, weaken the competitive pathway and reduce the depolymerization of mcl-PHA. Specifically, after various genetic modifications, the engineered *E. coli* ∆4D (ACP-SCLAC) preferentially used xylose to secrete fatty acid and acetic acid, while the engineered *P. putida* KTΔABZF (p2-a-J) could use glucose for its own growth and reproduction, and then used fatty acid and acetic acid to achieve the production of mcl-PHA. At the same time, the toxic effect of acetic acid on the *E. coli* cells was relieved to a large extent. Experimental results verified this design: as shown in Figure 3 of this study, *P. putida* KT∆ABZF (p2-a-J) produced 1.80 g/L mcl-PHA using mixed substrates (10 g/L glucose, 5 g/L acetic acid, 3 g/L octanoic acid and 2 g/L p-coumaric acid), which indicated that the engineered *P. putida* KT∆ABZF (p2-a-J) could produce mcl-PHA in the presence of acetic acid and fatty acids and also demonstrated its detoxification effect; Figures 4C,D in the study demonstrated the ability of *E. coli* ∆4D (ACP-SCLAC) to secrete acetic acid and fatty acids from lignocellulosic hydrolysate with the titer of 2.32 and 0.69 g/L, respectively, indicating that it could supply *P. putida* KT∆ABZF (p2-a-J) nutrition.

The purpose of constructing the microbial consortium in this study was to use a cheap carbon source for mcl-PHA production, and the results showed (Figure 6) that the consortium was able to produce 1.02 g/L mcl-PHA using lignocellulosic hydrolysate, which was comparable with the titer from mixed sugars and was increased by 135.02% from lignocellulosic hydrolysate than our previously constructed consortium (Liu et al., 2020). Petroleum-based plastics are already produced on a very mature scale, with a minimum selling price of about $1 445 per ton (Kim et al., 2022). Currently, the cost of the production of mcl-PHA by microorganisms is still higher than the conventional petroleum-based plastics. However, the use of microbial plants for mcl-PHA production has a broader prospect in view of the increasing crude oil prices and a series of environmental and health problems coming with petroleum-based plastics. There are few published literatures on the techno-economic analysis of mcl-PHA as far as we know, but there are some reports discussing scl-PHA, which has already been industrially produced and is sold at a minimum price of about $4 000 per ton currently (Wang et al., 2022). Meanwhile, current production of mcl-PHA is mainly on laboratory scale because of its higher production cost as well as lower production, compared with scl-PHA. The substrate is considered to be the key factor in the final production cost in the fermentation production of PHA, accounting for about 50% of the total cost (Kim, 2000; Nitzsche et al., 2016). The main substrates reported to be used for the production of mcl-PHA with microorganisms include p-coumaric acid, glucose, propionate, nonanoic acid (one of the fatty acids), and xylose, etc. (Davis et al., 2015; Zhuang and Qi, 2019; Salvachúa et al., 2020b; Zhu et al., 2021), all of which are high-cost substrates. In contrast, the lignocellulosic hydrolysate used in this study is generated from waste corn stover by pretreatment, thus would significantly reduce the substrate cost, which is also considered to be an excellent substrate for many other products (Salvachúa et al., 2016). As noticed, there is still space for the production improvement of the two-species microbial consortium, which requires improvement of corn stover pretreatment methods, further modification of engineered strains, and optimization of fermentation conditions to achieve the purpose of mass production of high value-added products with low substrate.

## 4 Conclusion

In this study, we reconstructed and optimized a previously developed microbial consortium consisting of engineered *P. putida* and *E. coli*, which can produce mcl-PHA from lignocellulosic hydrolysate. Heterologous expression of the gene encoding laccase from *S. azureus* resulted in the engineered *E. coli* ∆4D (ACP-SCLAC), which could produce 3.38 g/L of acetic acid using 10 g/L xylose as substrate, indicating that the acetic acid production capacity was not reduced by the metabolic burden imposed by the dual expression vector. At the same time, 0.67 g/L of FFAs was obtained, which was 48.89% higher than that in *E. coli* ∆4D. In the engineered strain *P. putida* KT∆ABZF (p2-a-J), the *phaZ* gene encoding PHA depolymerase and the *yqeF* gene were also knocked out in order to weaken fatty acid β-oxidation, resulting in a mcl-PHA titer of 3.98 g/L using 10 g/L glucose and 5 g/L octanoic acid as substrates, which was 1.75 times higher than that of the wild-type *P. putida* KT2440. The microbial consortium consisting of these above two engineered bacteria had good interspecies communication, and the consortium could efficiently produce an mcl-PHA titer of 1.64 g/L using a mixed carbon source consisting of 10 g/L glucose and 10 g/L xylose. Furthermore, the consortium could accumulate 1.02 g/L of mcl-PHA using lignocellulosic hydrolysate containing 10.50 g/L glucose and 10.21 g/L xylose, which had a competitive advantage (Table 2). In all, the successful reconstruction of the microbial consortium based on the concept of “nutrient supply-detoxification” can be used as a basis for further process development to produce high value-added compounds such as mcl-PHA from lignocellulosic biomass.

**TABLE 2:** Research on artificial consortia for PHA synthesis.

Artificial consortia	Substrate	Type of PHA	Titer (g/L)	Yield (g/g)	Production ratio (g/L/h)	References
*P. putida* KT2440 + *E. coli* MG1655	Glucose + Xylose	mcl-PHA	1.64 ± 0.10	0.08	0.03	This study
*P. putida* KT2440 + *E. coli* MG1655	Glucose + Pretreatments of corn stover	mcl-PHA	1.02 ± 0.01	0.06	0.02	This study
*P. putida* KT2440 + *E. coli* MG1655	Pretreatments of corn stover	mcl-PHA	0.65 ± 0.01	0.05	0.01	The study
*P. putida* KT2440 + *E. coli* MG1655	Glucose + Xylose	mcl-PHA	1.32 ± 0.03	0.07	0.02	Zhu et al. (2021)
*P. putida* KT2440 + *E. coli* MG1655	Glucose + Xylose	mcl-PHA	0.54 ± 0.03	0.03	0.01	Liu et al. (2020)
*P. putida* KT2440 + *E. coli* MG1655	Corn stover hydrolysate	mcl-PHA	0.43 ± 0.02	0.02	0.01	Liu et al. (2020)
*S. elongatus* cscB + *P. putida* cscAB	CO_2_	mcl-PHA	0.16 ± 0.04	N.A.	<0.01	Loewe et al. (2017)
*C. necator* DSM 428 + *P. citronellolis* NRRL B-2504	Apple pulp waste	P (3HB) and mcl-PHA	1.85 ± 0.03	0.11	0.04	Rebocho et al. (2020)
*S. degradans* 2-40 + *B. cereus*	Xylan	scl-PHA	0.27	N.A.	<0.01	Sawant et al. (2017)
*R. eutropha* H16 + *B. subtilis* 5119	Sugarcane sugar	scl-PHA	2.30	0.08	0.02	Bhatia et al. (2018)
*A. hydrophila* ATCC7966 + *A. junii* BP25	Acetic acid + butyric acid	scl-PHA	2.64	N.A.	0.01	Anburajan et al. (2019)
*C. necator* IPT 026 + *X. campestris* IBSBF 1867	Palm oil	scl-PHA	6.43	0.05	0.05	Rodrigues et al. (2019)

Values were calculated based on visible data of the original paper with unified to two decimal places. The yield is the ratio of the final PHA titer to the substrate concentration consumed. The production ratio is the titer of the PHA during a specific period. The data in the “Titer” column without the standard error added is not available in the original paper. N.A., not available in the original paper.

## Data Availability

The original contributions presented in the study are included in the article/Supplementary Material, further inquiries can be directed to the corresponding author.
