# Supplementary material for: Reconstruction and optimization of a Pseudomonas putida-Escherichia coli microbial consortium for mcl-PHA production from lignocellulosic biomass
Source: Front Bioeng Biotechnol. 2022 Oct 19;10:1023325. doi: 10.3389/fbioe.2022.1023325 (PMC9626825; doi:10.3389/fbioe.2022.1023325)
Supplement: Supplementary file 1 [file DataSheet1.pdf]

Supplementary Material

Table S1. Strains and plasmids are used in this work.

Name	Relevant characteristics	Source
Escherichia coli		
DH5α	F⁻ ϕ80d *lac*ZΔM15Δ(*lac*ZYA-*arg*F)U169 *end*A1 *rec*A1 *hsdR*17 (r_k^-, m_k^+) *sup*E44λ-*thi*-1 *gyrA*96 *rel*A1 *pho*A	TransGen Biotech
S17-1	*RP4-2(Km::Tn7, Tc::Mu-1) pro-82, LAMpir recA1 thiE1 hsdR17, creC510*	Lab stock
MG1655	*E. coli* K-12 F⁻λ⁻*ilv*G⁻*rfb*-50*rph*-1	Lab stock
Δ4D	*E. coli* MG1655, *ΔptsG, ΔmanZ, ΔatpFH, ΔenvR, ΔfadD*	Lab stock
E. coli Δ4D (ACP)	*E. coli* Δ4D harboring pET-T_3-ACP	Lab stock
E. coli Δ4D (*tesA*)	*E. coli* Δ4D harboring pET-T_3- *tesA*	This study
E. coli Δ4D (*fabZ*)	*E. coli* Δ4D harboring pET-T_3- *fabZ*	This study
E. coli Δ4D (*fabD*)	*E. coli* Δ4D harboring pET-T_3- *fabD*	This study
E. coli Δ4D (AD)	*E. coli* Δ4D harboring pET-T_3-ACP-*fabD*	This study
Pseudomonas putida		
KT2440	Wild-type	Lab stock
KT2440(p2)	*P. putida* KT2440 harboring pBBR1MCS-2	Lab stock
KTΔAB	*P. putida* KT2440, *ΔfadA, ΔfadB*	Lab stock
KT2400ΔZ	*P. putida* KT2440 knocked out *phaZ*	This study
KTΔABZ	*P. putida* KTΔAB knocked out *phaZ*	This study
KTΔABZF	*P. putida* KTΔABZ knocked out *yeqF*	This study
KTΔABZFJ	*P. putida* KTΔABZF knocked out *paaJ*	This study

KTΔABZFJT	*P. putida* KTΔABZFJ knocked out *tctA*	This study
KTΔABZFJ	*P. putida* KTΔABZF knocked out *paaJ*	This study
KTΔABZF (p2)	*P. putida* KTABZF harboring pBBR1MCS-2	This study
KTΔABZF (p2-a-J)	*P. putida* KTΔABZF harboring vector p2$_{T3}$-acs-phaJ	This study
KTΔABZF (p2$_{T3}$-C1C2)	*P. putida* KTΔABZF harboring vector p2$_{T3}$-C1C2	This study
KTΔABZF (p2$_{tac}$-C1C2)	*P. putida* KTΔABZF harboring vector p2$_{tac}$-C1C2	This study
KTΔABZF (p2$_{T3}$-SCLAC)	*P. putida* KTΔABZF harboring vector p2$_{T3}$-SCLAC	This study
KTΔABZF (p2$_{tac}$-SCLAC)	*P. putida* KTΔABZF harboring vector p2$_{tac}$-SCLAC	This study
KTΔABZF (p2-a-J-C1C2)	*P. putida* KTΔABZF harboring vector p2$_{T3}$-acs-phaJ-C1C2	This study
Plasmids		
pET28a	The expression vector for *E. coli*	Lab stock
pBBR1MCS-5	Expression vector for a wide host	Lab stock
pBBR1MCS-2	The expression vector for *P. putida*	Lab stock
pK18mobsacB	Helper knockout plasmid vector	Lab stock
pET-ACP	T_3 promoter, RBS, and ACP inserted into pET28a	Lab stock
pET-*tesA*	T_3 promoter, RBS, and *tesA* inserted into pET28a	This study
pET-*fabZ*	T_3 promoter, RBS, and *fabZ* inserted into pET28a	This study
pET-*fabD*	T_3 promoter, RBS and *fabD* inserted into pET28a	This study
pET-ACP-*fabD*	T_3 promoter, RBS and *fabD* inserted into pET-T_3-ACP	This study
pET-ACP-*tesA*-*fabD*	T_3 promoter, RBS and *tesA* inserted into pET-T_3-ACP-*fabD*	This study
pET-ACP-*fabZ*-*fabD*	T_3 promoter, RBS and *fabZ* inserted into pET-T_3-ACP-*fabD*	This study
pET-ACP-*tesA*-*fabZ*-*fabD*	T_3 promoter, RBS and *fabZ* inserted into pET-T_3-ACP-*tesA*-*fabD*	This study

p5-T_3-SCLAC	T_3 promoter, RBS and SCLAC inserted into pBBR1MCS-5	This study
pK18-*phaZ*	helper knockout phaZ plasmid vector	This study
pK18-*yqeF*	helper knockout *yqeF* plasmid vector	This study
pK18-*paaJ*	helper knockout *paaJ* plasmid vector	This study
pK18-*tctA*	helper knockout *tctA* plasmid vector	This study
p2-a-J	*acs* and *phaJ* inserted into pBBR1MCS-2	This study
$p2_{T3}$-C1C2	T3 promoter, *phaC1* and *phaC2* inserted into pBBR1MCS-2	This study
$p2_{tac}$-C1C2	Tac promoter, *phaC1* and *phaC2* inserted into pBBR1MCS-2	This study
$p2_{T3}$-SCLAC	T3 promoter and SCLAC inserted into pBBR1MCS-2	This study
$p2_{tac}$-SCLAC	Tac promoter and *Sclac* inserted into pBBR1MCS-2	This study
p2-a-J-$_{tac}$-C1C2	*acs*, *phaJ*, Tac promoter, *phaC1* and *phaC2* inserted into pBBR1MCS-2	This study

Table S2. Primers used for the construction of recombinant plasmids.

Primers[a]	Sequence (5'→3')
E. coli	
T_3-RBS-f1	CCAAGCGCGCAATTAACCCTCACTAAAGGGAACAAAAGCTGATACGTATTTAAATCAGG
T_3-RBS-f2	TTTCACACAGGAAACAGCTATGACCATGATTACGCCAAGCGCGCAATTAAC
pET-T_3-RBS-f	CGTCCGGCGTAGAGGATCGAGATCTTTTCACACAGGAAACAGC
RBS-*tesA*-f	GCTGATACGTATTTAAATCAGGAGTGGAAATGATGAACTTCAACAATGTTTTCCGCTGGC
pET-*tesA*-r	GCAAGCTTGTCGACGGAGCTCGAATTCTTATGAGTCATGATTTACTAAAGG
RBS-*fabZ*-f	GCTGATACGTATTTAAATCAGGAGTGGAATTGACTACTAACACTCATACTCTGCAG
pET-*fabZ*-r	CAAGCTTGTCGACGGAGCTCGAATTCTCAGGCCTCCCGGCTACGAG
RBS-*fabD*-f	GCTGATACGTATTTAAATCAGGAGTGGAAATGACGCAATTTGCATTTGTGTTCCC
pET-*fabD*-r	CAAGCTTGTCGACGGAGCTCGAATTCTTAAAGCTCGAGCGCCGCTG
XbaI-*tesA*-f	GCTCTAGATTTCACACAGGAAACAGCTATGACC
NheI-*tesA*-r	CTAGCTAGCTTATGAGTCATGATTTACTAAAGGCTG
NheI-*fabZ*-f	CTAGCTAGCTTTCACACAGGAAACAGCTATGACCATG
BamHI-*fabZ*-r	CGGGATCCTCAGGCCTCCCGGCTACGA
BamHI-*fabD*-f	CGGGATCCTTTCACACAGGAAACAGCTATGACCATGATTACGCCAAGCG
NotI-*fabD*-r	ATAAGAATGCGGCCGCTTAAAGCTCGAGCGCCG
pET28a-f	GAGCCCGATCTTCCCCATCGGTGATGTC
pET28a-r	CCGGATATAGTTCCTCCTTTCAGCAAAAAACCCCTCAAG
RBS-ESC-f	GTATTTAAATCAGGAGTGGAAATGGATCGCCGCGGTTTTAATCGTCGTG
p5-ESC-f	CCCTCACTAAAGGGAACAAAAGCTGGGTACCATACGTATTTAAATCAGGAGTGGAAATG
p5-ESC-r	CTAGTGGATCCCCCGGGCTGCAGGAATTCTTAATGGTGATGGTGATGATGATGTTC
p5-f	GTGAGTTAGCTCACTCATTAGGCACCCCAGG

p5-r	CACGACGTTGTAAAACGACGGCCAGTGAG
P. putida	
phaZ up-f	CAGGAAACAGCTATGACATGATTACGAATTCATGTTCAAGAGCAACCCGCTGACCCGCC
phaZ up-r	CCTGTCAGGCCGCAGCTGTTGCACGTGACTCTTGGGTGAAGTAAAC
phaZ down-f	GTTTACTTCACCCAAGAGTCACGTGCAACAGCTGCGGCCTGACAGG
phaZ down-r	CGACGGCCAGTGCCAAGCTTCGGTGTTGAACAGCTCCTTGAC
yqeF up-f	CAGCTATGACATGATTACGAATTCGAAGCCGAAGGCCGCCGTGATGC
yqeF up-r	GAAGAGGCCGGGCCTGCTTGAATCAAGTTGTTGTTCTCCGTGCAATG
yqeF down-f	CATTGCACGGAGAACAACAACTTGATTCAAGCAGGCCCGGCCTCTTC
yqeF down-r	CGACGGCCAGTGCCAAGCTTAGAAGCTGATCGCCACGTACTTC
paaJ up-f	AACAGCTATGACATGATTACGAATTCCCGATGCCTTGATCATCGACGC
paaJ up-r	TCATTCACGCCGGAGGCATTTCAGCCGTTTCCGGCATC
paaJ down-f	GATGCCGGAAACGGCTGAAATGCCTCCGGCGTGAATGA
paaJ down-r	GTAAAACGACGGCCAGTGCCAAGCTTCATATACGGCTGCGTTCAG
tctA up-f	CAGCTATGACATGATTACGAATTCATTTGCATTTCACTTGAGCAATCCC
tctA up-r	CGAACAATGGCAACGACCGATTCAGTTCTCCAGTACGCTC
tctA down-f	GAGCGTACTGGAGAACTGAATCGGTCGTTGCCATTGTTCG
tctA down-r	CGACGGCCAGTGCCAAGCTTGTACCCCGTCACCCAGCAG
acs-phaJ-f	GGGGTACCATGCCCCAGCCAAGCTACACC
acs-phaJ-r	CCCAAGCTTTCAGCTCGCCACAAAGTTC
phaC1-tac-f	GGGAACAAAAGCTGGGTACCCCCGTTCTGGATAATGTTTTTTGC
phaC1-tac-r	GCTCATCGTTGTTCTTGTTACTCATCTAGTATTCCACTCCTGATTTAAATACGTATG
phaC1-f	CATACGTATTTAAATCAGGAGTGGAATACTAGATGAGTAACAAGAACAACGATGAGC
phaC1-r	CCTTTGGCCGGTTTGTCTGTCATTCAACGCTCGTGAACGTAGG

phaC2-f	CCTACGTTCACGAGCGTTGAATGACAGACAAACCGGCCAAAGG
phaC2-r	CGACTGAGCCTTTCGTTTTATTTGATTCATCGGGTCAGCACGTAGG
RBS-C1-f	CACTAAAGGGAACAAAAGCTGGGTACCATACGTATTTAAATCAGGAGTGGAATAC
RBS-C1-r	CCGCTCTAGAACTAGTGGATCCTCAACGCTCGTGAACGTAGGTG
C1C2-f	CTACGTTCACGAGCGTTGAGGATCCATGACAGACAAACCGGCCAAAG
C1C2-r	GCGGTGGCGGCCGCTCTAGAACTAGTTCATCGGGTCAGCACGTAG
SCLAC-tac-f	CGAATTCCTGCAGCCCGGGGGATCCCCCGTTCTGGATAATGTTTTTTG
SCLAC-tac-r	GGTTGAAGCCGCGGCGGTCCATCTAGTATTCCACTCCTGATTTAAATACG
SCLAC-f	CGTATTTAAATCAGGAGTGGAATACTAGATGGACCGCCGCGGCTTCAACC
SCLAC-r	CTATAGGGCGAATTGGAGCTCTCAATGGTGATGGTGATGATGGTGC
RBS-SCLAC-f	CACTAAAGGGAACAAAAGCTGGGTACCATACGTATTTAAATCAGGAGTGGAATAC
RBS-SCLAC-r	GCTGCAGGAATTCGATATCAAGCTTTCAATGGTGATGGTGATGATGGTGC
phaJ-C1C2-f	GCCGAACTTTGTGGCGAGCTGAAAGCTTTTTCACACAGGAAACAGCTATG
p2-f	ATGGCATTCGAAACCATCCTGTTGGACATCCACGGCAAGGTTGG
p2-r	TCAACGGTCCTTGAACTGTGCCTCACGCTTGGCGATG

[a]Restriction sites are shown in underline.

Table S3. Monomer compositions of the mcl-PHA synthesized by the engineered *P. putida*.

Strains	Substrates	Mcl-PHA titer (g/L)	CDW (g/L)	Mcl-PHA Content (wt%)	Monomer compositions (%)				
					3HHx (C6)	3HO (C8)	3HD (C10)	3HDD (C12)	3HTD (C14)
**Gene knockout strains**									
KT2440	10 g/L glucose and 5 g/L octanoic acid	2.27 ± 0.08	3.82 ± 0.11	59.43 ± 3.76	6.66 ± 0.23	78.04 ± 0.05	10.43 ± 0.07	4.86 ± 0.11	NA
KT2440ΔZ		3.37 ± 0.16	4.84 ± 0.06	76.29 ± 4.15	4.42 ± 0.56	78.60 ± 1.60	7.86 ± 0.66	3.77 ± 0.22	5.35 ± 0.17
KTΔAB		1.31 ± 0.33	2.12 ± 0.38	61.38 ± 4.49	1.50 ± 0.23	81.81 ± 5.40	7.10 ± 2.56	3.80 ± 0.98	5.78 ± 1.64
KTΔABZ		2.63 ± 0.31	2.94 ± 0.29	81.48 ± 4.51	1.64 ± 0.42	88.12 ± 2.54	4.24 ± 0.60	2.39 ± 0.49	3.60 ± 1.03
KTΔABZF		3.62 ± 0.40	4.5 ± 0.17	78.00 ± 1.42	1.56 ± 0.11	87.89 ± 0.79	4.52 ± 0.13	2.59 ± 0.24	3.44 ± 0.51
KTΔABZFJ		2.12 ± 0.30	2.46 ± 0.01	85.89 ± 1.07	2.15 ± 0.20	87.81 ± 1.43	2.82 ± 0.08	5.84 ± 1.56	1.38 ± 0.00
KTΔABZFJT		1.56 ± 0.01	2.41 ± 0.06	64.53 ± 5.76	5.91 ± 0.31	39.56 ± 5.69	31.12 ± 4.33	6.17 ± 0.66	17.25 ± 0.93
**Gene overexpression**									
KTΔABZF (p2)	10 g/L glucose and 5 g/L octanoic acid	1.25 ± 0.16	1.88 ± 0.19	66.29 ± 2.36	4.02 ± 0.39	85.18 ± 0.60	3.77 ± 0.22	3.28 ± 0.02	3.75 ± 0.24
KTΔABZF (p2-a-J)		3.98 ± 0.34	4.33 ± 0.35	91.96 ± 0.31	7.99 ± 0.66	86.69 ± 0.13	1.73 ± 0.09	2.18 ± 0.56	4.14 ± 0.43
KTΔABZFJ (p2-a-J)		3.15 ± 0.39	3.65 ± 0.16	86.39 ± 8.48	9.99 ± 0.54	82.72± 1.72	1.74 ± 0.11	3.40 ± 0.16	5.83 ± 0.88
KTΔABZF ($p2_{tac}$-C1C2)		3.69 ± 0.71	3.81 ± 0.75	96.77 ± 0.53	5.80 ± 0.60	79.23 ± 0.26	5.40 ± 0.53	5.67 ± 0.06	3.90 ± 0.14
KTΔABZF ($p2_{T3}$-C1C2)		0.82 ± 0.23	1.07 ± 0.18	76.12 ± 8.64	7.89 ± 0.92	73.18 ± 1.50	5.73 ± 0.28	8.27 ± 0.43	4.92 ± 0.43
KTΔABZF (p2-a-J-C1C2)		1.04 ± 0.02	2.07 ± 0.08	50.15 ± 2.65	NA	74.14 ± 0.55	8.79± 1.17	8.12 ± 0.02	8.95 ± 0.60
**Heterologous expression**									
KT2440	10 g/L glucose	0.53 ± 0.02	3.02 ± 0.1	17.54 ± 1.16	NA	7.98 ± 1.21	33.34 ± 1.50	19.68 ±0.84	39.00 ± 1.88
KTΔABZF		1.42 ± 0.04	4.22 ± 0.07	33.7 ± 0.3	6.71 ± 0.03	8.53 ± 0.26	43.44 ± 0.01	19.48 ±0.08	21.83 ± 0.20

KTΔABZF (p2)		0.61 ± 0.02	2.75 ± 0.22	22.17 ± 0.97	9.67 ± 0.43	7.25 ± 0.25	30.66 ± 1.75	22.91 ±0.67	29.51 ± 0.39
KTΔABZF (p2$_{tac}$-SCLAC)		0.81 ± 0.01	2.82 ± 0.23	28.73 ± 2.62	7.85 ± 0.04	7.06 ± 0.14	38.99 ± 0.11	21.47 ±0.32	24.62 ± 0.31
KTΔABZF (p2$_{T3}$-SCLAC)		1.14 ± 0.07	4.00 ± 0.02	28.53 ± 1.16	6.36 ± 0.42	7.66 ± 0.32	40.50 ± 1.37	21.68 ±0.44	23.79 ± 1.07
KT2440	10 g/L glucose and 2 g/L p-CA	0.32 ± 0.03	1.57 ± 0.07	20.34 ± 0.88	20.92 ±1.81	26.21 ± 2.52	12.96 ± 1.15	11.31 ±1.18	28.59± 3.00
KTΔABZF		0.81 ± 0.11	1.92 ± 0.19	42.19 ± 6.64	9.96 ± 4.27	18.14 ± 1.22	45.16 ± 3.72	10.05 ±1.06	16.69 ± 0.95
KTΔABZF (p2)		0.54 ± 0.02	1.7 ± 0.06	31.71 ± 2.16	20.28 ±5.71	24.15± 4.25	21.37 ± 1.51	13.80 ±3.84	23.09 ± 3.93
KTΔABZF (p2$_{tac}$-SCLAC)		1.05 ± 0.19	2.12 ± 0.13	49.39 ± 5.79	6.77 ± 0.74	6.87 ± 0.08	56.75 ± 0.49	13.52 ±0.78	16.09 ± 0.43
KTΔABZF (p2$_{T3}$-SCLAC)		0.85 ± 0.02	2.06 ± 0.17	41.19 ± 1.44	3.43± 0.08	13.09 ± 0.23	44.70 ± 1.14	15.56 ±0.07	23.22 ± 1.36
**strains**									
KT2440	10 g/L glucose, 5 g/L acetic acid, 3 g/L octanoic acid and 2 g/L p-CA	1.04 ± 0.14	1.57 ± 0.17	66.41 ± 1.86	13.20± 0.27	70.61 ± 1.81	4.87 ± 1.11	4.73 ± 0.37	6.58 ± 0.06
KTΔABZF		1.54 ± 0.08	1.96 ± 0.03	78.67 ± 5.58	8.54 ± 2.35	74.07 ± 0.04	6.85 ± 2.12	5.09 ± 0.63	5.45 ± 0.44
KTΔABZF (p2)		0.96 ± 0.01	1.36 ± 0.04	70.49 ± 2.95	6.06 ± 1.42	79.57 ± 1.90	3.02 ± 0.47	5.07 ± 0.20	6.27 ± 0.19
KTΔABZF (p2$_{tac}$-SCLAC)		1.16 ± 0.02	1.8 ± 0.06	64.62 ± 0.91	6.25 ± 0.21	73.52 ± 0.88	6.48 ± 0.34	6.53 ± 0.23	7.21 ± 0.52
KTΔABZF (p2$_{T3}$-SCLAC)		0.74 ± 0.18	1.59 ± 0.28	46.33 ± 3.01	9.63 ± 3.83	68.60 ± 5.02	6.97 ± 0.42	7.65 ± 0.06	7.15 ± 1.55
KTΔABZF (p2$_{tac}$-C1C2)		1.96 ± 0.14	2.47 ± 0.14	79.24 ± 1.22	8.10 ± 0.73	71.89 ± 2.03	7.93 ± 2.30	6.76 ± 0.11	5.32 ± 0.35
KTΔABZF (p2-a-J)		1.80 ± 0.20	2.37 ± 0.32	76.01 ± 1.69	6.06 ± 0.00	78.57 ± 1.04	4.46 ± 1.25	6.01 ± 0.12	4.90 ± 0.33
KTΔABZF (p2-a-J-C1C2)		1.06 ± 0.36	1.75 ± 0.29	60.16 ±10.52	2.14 ± 1.00	76.04 ± 0.88	3.42 ± 0.07	10.19± 1.14	8.20 ± 1.10

Table S4. The consumption of sugars by the engineered *E. coli* using corn straw pretreatment.

Engineered strains	Initial concentration (g/L)		Final concentration (g/L)	
	Glucose	xylose	Glucose	xylose
E. coli Δ4D	1.29 ± 0.08	20.25 ± 1.31	0	17.66 ± 2.07
E. coli Δ4D (ACP)	1.35 ± 0.01	21.35 ± 0.08	0	16.49 ± 0.01
E. coli Δ4D (ACP-SCLAC)	1.00 ± 0.29	21.35 ± 0.44	0	16.77 ± 1.18

Table S5. The utilization of sugars in lignocellulose hydrolysate by the *P. putida-E. coli* consortium.

Fermentation medium	Initial concentration (g/L)		Final concentration (g/L)		Yield (g/g sugar)
	Glucose	xylose	Glucose	xylose	
M1-1	0.45 ± 0.02	9.44 ± 1.06	0	2.75 ± 0.40	0.037
M1-1+	10.50 ± 1.58	10.21 ± 0.19	0	2.98 ± 0.72	0.054
M9-1	2.03 ± 0.14	22.56 ± 1.24	0	10.99 ± 0.00	0.046
M9-1+	10.31 ± 0.95	21.80 ± 0.48	0	17.20 ± 0.92	0.038
Enzymatic digest solution	9.29 ± 0.55	8.59 ± 0.74	1.17 ± 0.30	6.17 ± 0.31	0.043

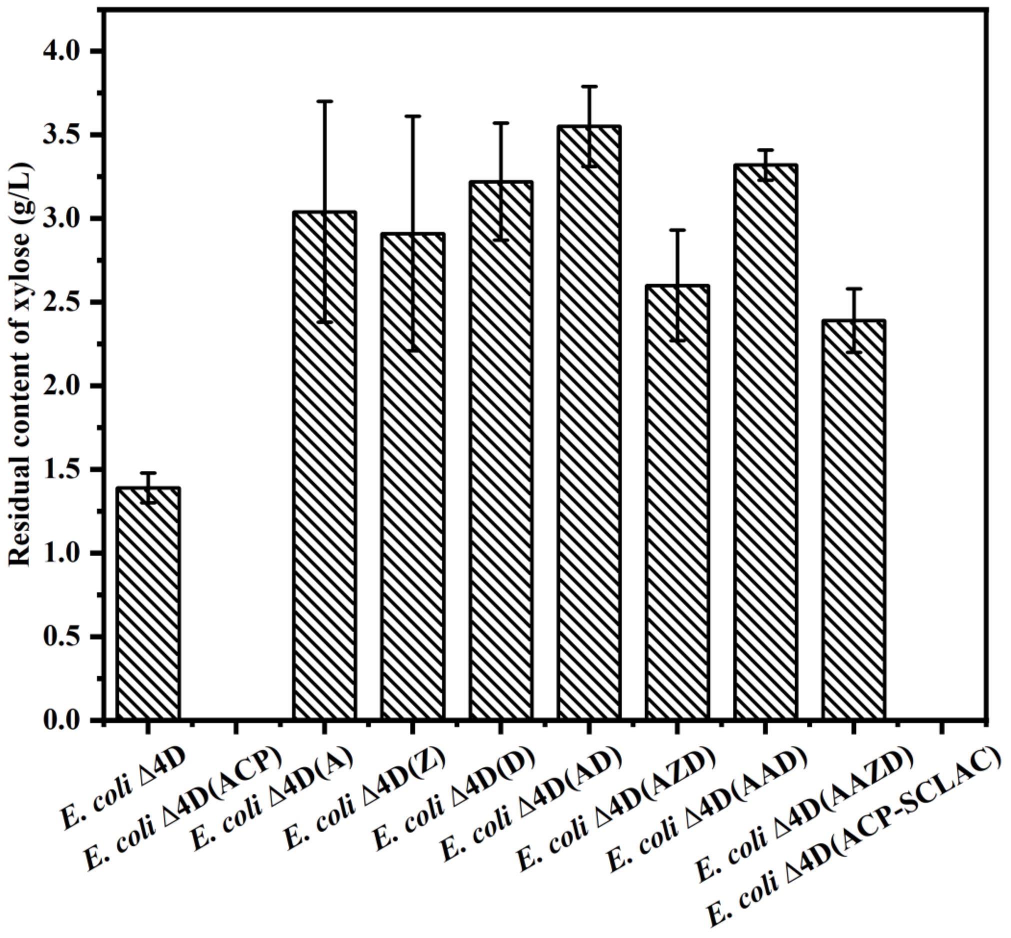

Supplementary Figure 1. The residual contents of xylose (g/L) by engineered different *E. coli* for 64 hours.

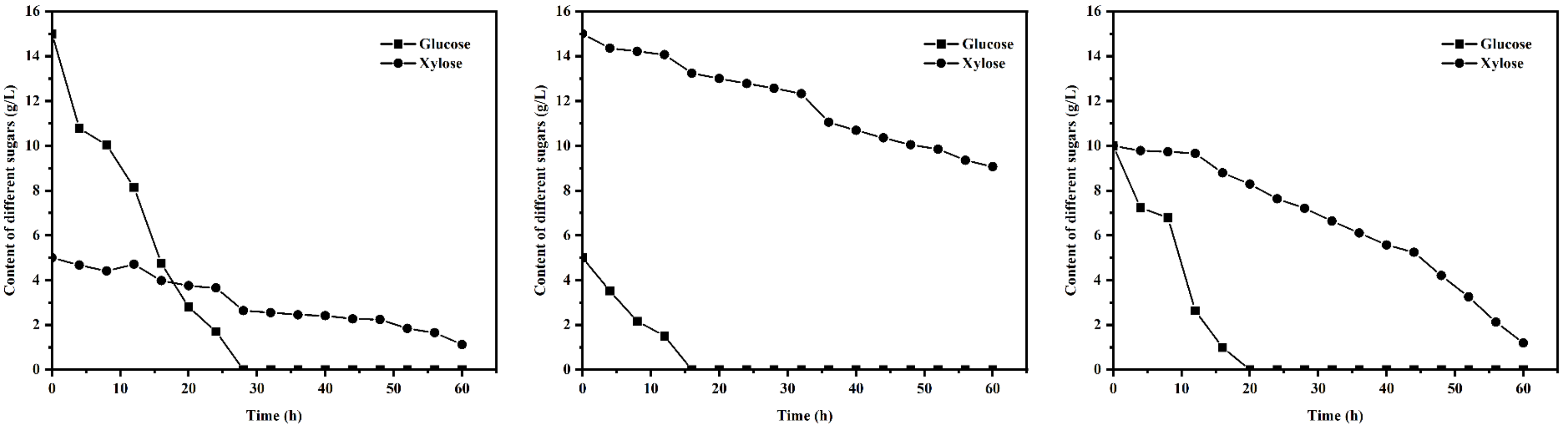

Supplementary Figure 2. The residual contents of glucose and xylose under different mixed sugar ratios by the microbial consortium.

(A) The ratio of glucose and xylose was 3:1; (B) The ratio of glucose and xylose was 1:3; (C) The ratio of glucose and xylose was 1:1.
